# A training strategy for hybrid models to break the curse of dimensionality

**DOI:** 10.1371/journal.pone.0274569

**Published:** 2022-09-15

**Authors:** Moein E. Samadi, Sandra Kiefer, Sebastian Johaness Fritsch, Johannes Bickenbach, Andreas Schuppert

**Affiliations:** 1 Institute for Computational Biomedicine, RWTH Aachen University, Aachen, Germany; 2 Joint Research Center for Computational Biomedicine, RWTH Aachen University, Aachen, Germany; 3 Max Planck Institute for Software Systems, Saarland Informatics Campus, Saarbrücken, Germany; 4 Department of Intensive Care Medicine, University Hospital RWTH Aachen, Aachen, Germany; 5 Jülich Supercomputing Centre, Forschungszentrum Jülich, Jülich, Germany; Fuzhou University, CHINA

## Abstract

Mechanistic/data-driven hybrid modeling is a key approach when the mechanistic details of the processes at hand are not sufficiently well understood, but also inferring a model purely from data is too complex. By the integration of first principles into a data-driven approach, hybrid modeling promises a feasible data demand alongside extrapolation. In this work, we introduce a learning strategy for tree-structured hybrid models to perform a binary classification task. Given a set of binary labeled data, the challenge is to use them to develop a model that accurately assesses labels of new unlabeled data. Our strategy employs graph-theoretic methods to analyze the data and deduce a function that maps input features to output labels. Our focus here is on data sets represented by binary features in which the label assessment of unlabeled data points is always extrapolation. Our strategy shows the existence of small sets of data points within given binary data for which knowing the labels allows for extrapolation to the entire valid input space. An implementation of our strategy yields a notable reduction of training-data demand in a binary classification task compared with different supervised machine learning algorithms. As an application, we have fitted a tree-structured hybrid model to the vital status of a cohort of COVID-19 patients requiring intensive-care unit treatment and mechanical ventilation. Our learning strategy yields the existence of patient cohorts for whom knowing the vital status enables extrapolation to the entire valid input space of the developed hybrid model.

## Introduction

By learning from data, Machine Learning (ML) allows to model complex systems in which the mechanisms controlling the system are poorly understood [1]. Such challenges appear particularly in medical and biomedical contexts [2, 3]. Here, ML has become one of the most practicable tools for building predictive models [4, 5]. However, the predictions obtained with ML methods are only reliable within the convex hull of the given training data [6, 7]. Extrapolation, i.e., accurate prediction beyond the convex hull of the given training data, is conceptually impossible without further enhancement of the machinery [7–9].

Another drawback of ML methods is that they suffer from the curse of dimensionality (COD) [10]. The COD refers to the high demand for training data, which is usually exponential in the complexity of the model [11, 12]. Towards breaking the COD, ML methods such as DNNs have been developed for specific classes of input-output (i-o) functions [13, 14]. However, also DNNs cannot offer a generic solution for all classes of functions that need to be approximated [15].

These drawbacks of pure ML methods hamper sufficient performance of predictive models in medical and biomedical applications [16, 17]. In particular, attempts to develop diagnostic and prognostic models for the individual patients suffering from Coronavirus disease 2019 (COVID-19) have shown a moderate performance alongside poor generalizability [18, 19]. The large number of potentially relevant features for prognosis and diagnosis of COVID-19 requires novel data analysis and predictive-model development methods [20]. The obtained models should be capable of making reliable predictions not only for multi-dimensional feature spaces but also from data of biased or small cohorts of patients [19, 21].

The aforementioned problems can be tackled by integrating *a priori* available knowledge of the system structure into ML learning processes, which is done in Structured Hybrid Models (SHMs) [22, 23]. SHMs can be realized by modular neural networks [24] with given connections among input features and network modules: each module of the first layer represents a known sub-process of the overall system and takes a subset of the input features as its input, every other module reads inputs from previous layers to compute its (intermediate) output. The final output modules then combine the precomputations to determine the overall output of the system. Each module of the network is represented either via known physics-based equations (white-boxes) or via an unknown black-box to be trained by ML methods. As attested by the COD, the complexity of each black-box module, which is the number of examples needed to determine the input-output function of the module, scales exponentially with the dimension of its input vectors [13]. By employing various black-box modules with fewer input variables, the overall complexity of an SHM is usually much lower than the respective complexity of pure ML methods where a single black-box deals with the entire input vector. This way, SHMs can serve as a framework to overcome the conceptual drawbacks of ML.

Particularly in process modeling, for example in chemical engineering [25–27], input-output relations are modeled as a composition of unknown black-box and known white-box modules. In such a hybrid structure, the overall model maps input data in ${\mathbb{R}}^{n}$ to outputs in ℝ. The number of inputs for each black-box module in the hybrid structure is typically much lower than the total number of inputs *n* to the network. It was shown in [22, 23] that all unknown functions of the black-box modules in a tree-structured network can be uniquely determined as long as the training data set is distributed (in a strong formal sense) around a *d*-dimensional manifold in ${\mathbb{R}}^{n}$ with sufficient differentiability, where *d* is a bound on the number of inputs to the black-box modules. In case *d* < *n*, the trained hybrid model can extrapolate resulting in the reduction of training data demand towards breaking the COD.

However, the superiority of hybrid models in terms of data-demand reduction and extrapolability as described in [22, 23] is based on the availability of densely distributed training data on low-dimensional subsets of ${\mathbb{R}}^{n}$. This property restricts applications of hybrid modeling to cases where highly correlated input-data distributions are available around low-dimensional manifolds within the input data space. In contrast to such controlled systems, observational data [28], such as in clinical data repositories, reflect mostly uncontrolled systems where the data distribution is not squeezed around low-dimensional manifolds. Moreover, observational data are often discrete or even binary.

In this work, we focus on binary data in order to provide a systematic extension of the hybrid models presented in [22, 23] towards hybrid models with randomly distributed training data within a binary feature space. This is meaningful for three reasons: first, binary hybrid models exhibit all characteristics regarding data-demand reduction and extrapolability of generic hybrid models without the specific numerical challenges of training on continuous data. Second, any monotonic discrete black-box function can be represented by a composition of binary black-box functions. This sequence indicates the generalizability of learning strategies on binary hybrid models to generic, discrete black-box-based models. Moreover, we expect learning strategies derived from binary models can further be generalized to even continuous feature spaces because discrete grid-based functions can be interpreted as local approximations for smooth continuous functions. Third, for binary data divided into training and test sets, any label assessment of unlabeled data points in the test set is always extrapolation, since any binary data point not contained in the training data lies outside the convex hull of the training data. Hence, the high prediction accuracy of a hybrid model for the test data, the out-of-sample forecast performance, is a direct indicator of the extrapolability of the hybrid model.

In this paper, we study classification tasks for binary labeled data represented by binary features. Given a set of data, the challenge is to use them to develop a model that accurately assesses labels of new unlabeled data. We present a learning strategy to compute a function that maps input features to output labels. We assume that the structure of the mapping between features and labels is known *a priori* and fits an SHM with an underlying tree structure. Our strategy uses graph-theoretic methods to deduce labels of new data points and to obtain a function that maps input features to output labels. It turns out that the classification efficiency of our hybrid model outperforms various supervised ML algorithms, namely Deep Neural Network (DNN), Support Vector Machine (SVM), Random Forest (RF), and Logistic Regression (LR). Additionally, our method shows the existence of small sets of data points for which knowing their labels allows for extrapolation to the entire feature space. Accordingly, our algorithm promises a lower training-data demand than sole data-driven methods.

In an application of our strategy, we have fitted a tree-structured hybrid model to the vital status of a cohort of COVID-19 patients requiring intensive-care unit treatment and mechanical ventilation. Our learning strategy yields the existence of patient cohorts for whom knowing the vital status enables extrapolation to the entire valid input space of the developed hybrid model.

The ability of hybrid models to extrapolate can boost applications of ML in medical and clinical research. In medical contexts, ML faces a variety of barriers. On the one hand, the patient-specific disease-driving mechanisms are often widely unexplored [17]. On the other hand, medical data repositories tend to be biased by specific patient cohorts and restricted in size, especially when compared to the reported data demands in DNN applications. Moreover, the pooling of clinical data from heterogeneous sources requires a high degree of administrative effort due to data privacy regulations. As a consequence, pure ML in medicine is currently focused on specific tasks such as time-series analysis and pattern recognition, where data is accessible from wearable devices and medical imaging technologies [16]. Therefore for clinical studies, the integration of knowledge and ML in a hybrid-model setting is essential, particularly for the development of predictive models that can make reliable predictions even outside the convex hull of the given data.

The paper is organized as follows. In the next section, we introduce tree-SHMs for binary classification. Then we present our learning strategy, comprising the *Conflict-Graph construction* and the *Label determination*, and we explain the graph-theoretic machinery we use. For the application, we then summarize the synthetic and COVID-19 data sources. Next, we discuss the classification efficiency and the training-data demand of our learning strategy on both the synthetic and the COVID-19 data. The paper ends with a conclusion and suggestions for future projects.

## Material and methods

### Model

This section introduces our hybrid model, which integrates available measurement data into *a priori* knowledge about the system. The input to our model consists of data obtained from measurements, e.g. physiological data. The data is represented as *d*-dimensional vectors for some d∈ℕ, where each entry corresponds to one feature that is assumed to be binary. So the input vectors are elements of {0, 1}^*d*^.

The general task is to learn an unknown function that assigns to each potential input vector its *label*, which can again be a 0 or a 1, depending on whether the data point belongs to the first or the second of the two classes that constitute the classification task. We use a given *training-data set* of input data and associated labels to learn the model. The training-data set covers only a small subset of the *d*-dimensional hypercube that contains all valid input vectors. From this information, we need to draw conclusions to also predict labels of unlabeled new data points.

**Fig 1:**
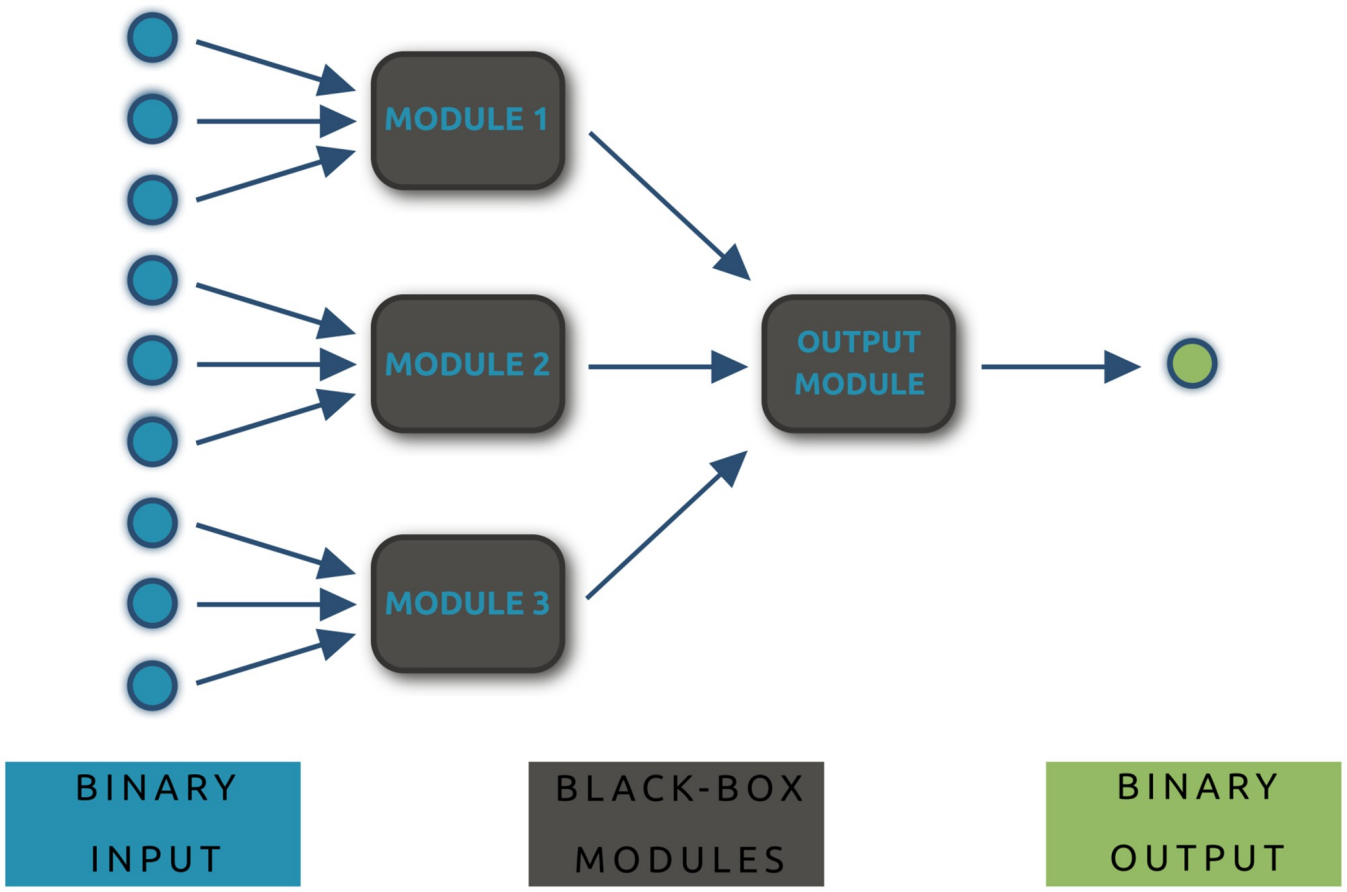
A tree-structured hybrid network. The network maps binary input variables *x* ∈ {0, 1}^9^ to binary outputs *y* ∈ {0, 1}. Three first-layer black-box modules each have separate input variables, and a single black-box module processes the partial outputs of the first layer to compute the overall output of the network, which can then be interpreted as a decision for one of the two considered classes.

In an SHM, see Fig 1 for a schematic example, mechanistic understanding of the underlying system is used to partially pre-determine the structure of a network which maps input variables to output values by combining several sub-computations performed in *modules*, where each module represents a separate sub-process within the overall system. In our setting, each network module is considered a black-box and will be trained using the available measurement data, up to redundant invariants [22, 23].

We furthermore assume that the hybrid network has a *tree structure* with two layers: in the first layer, independent black-box modules operate on separate input variables to complete sub-computations of the main classification. In the second layer, a black-box module processes the outputs of the modules from the previous layer towards the overall output of the network. Without loss of generality, we can assume that the entries of the input vectors are ordered according to the modules: that is, when there are *k* first-layer black-box modules, then the first first-layer black-box module has as its input the first *n*_1_ entries of the overall input, and for *i* ∈ {2, …, *k*}, the *i*-th module takes as input the *n*_*i*_ entries of the overall input starting at position $\sum_{j=1}^{i-1}n_{j}+1$.

In the described setting, the overall i-o relation can be represented as a *k*-dimensional orthotope (hyperrectangle), where *k* is the number of first-layer black-box modules of the network. Each cell of the orthotope represents a binary input vector, holding the corresponding output label of the vector (which is 0 or 1). Each axis of the orthotope corresponds to the possible inputs of one first-layer module and thus, the *i*-th axis has length $2^{n_{i}}$.

**Fig 2:**
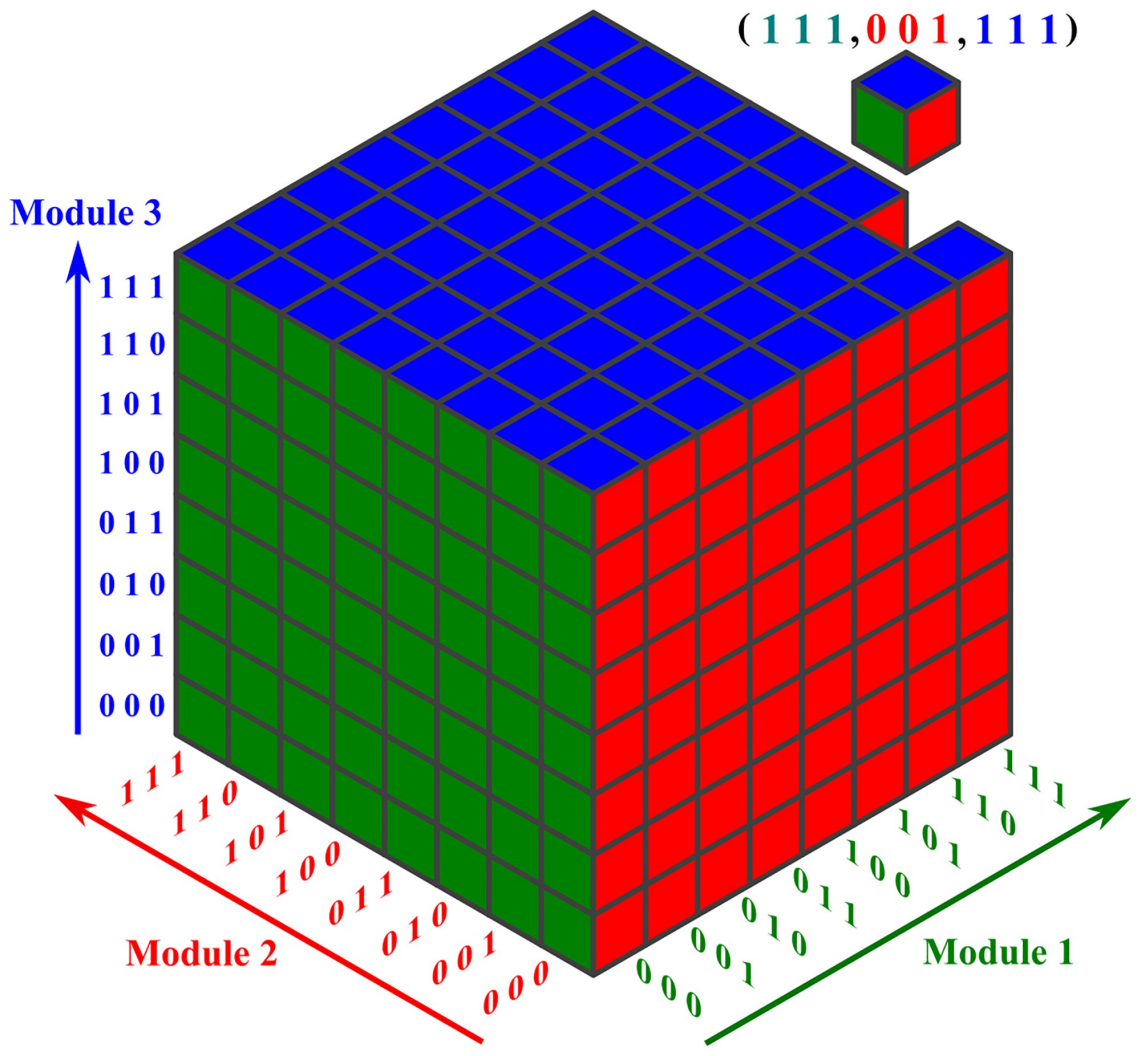
The orthotope for the network structure of Fig 1. The dimension of the orthotope is equal to the number of first-layer black-box modules of the tree-structured hybrid network of Fig 1. Each cell of the orthotope, characterized by three coordinates, represents an input data point holding the corresponding output label. Each intersection of a hyperplane with the orthotope holds input data with a constant input for a specific first-layer black-box module. For example, the uppermost horizontal blue slice of the orthotope illustrates all input vectors whose last three entries are 1, 1, 1.

An orthotope related to the network structure of Fig 1 is depicted in Fig 2. The network in Fig 1 is tree-structured with three first-layer black-box modules. The number of input variables for each module is 3. Therefore, the orthotope is a 3-dimensional hypercube with 2^3^ elements on each axis of the cube. So, altogether, the orthotope has 2^9^ cells, one for each valid binary input vector for the SHM. We equip each cell corresponding to a training-data point with a label that indicates the correct classification of the data point. The task is now to determine the i-o function, or, equivalently, to predict the labels for all cells in the orthotope.

### Learning strategy

Here we present our learning strategy for SHMs which is to perform a binary classification task for binary input data. Recall from the previous section that we assume 2-layer tree-shaped SHMs whose first layer, as well as the output layer, consist of black-box modules. The training strategy consists of two parts called the *Conflict-Graph construction* and the *Label determination*. Together, the two procedures serve to evaluate the effect of local modifications in the input on the overall output of the SHM. For some intuition, we give a detailed description of the two procedures first. We then provide the full algorithm in pseudocode.

We assume that our SHM has *k* first-layer black-box modules and denote for every *i* ∈ {1, …, *k*} by *n*_*i*_ the number of inputs to the *i*-th module. For example, the SHM of Fig 1 has *k* = 3 first-layer black-box modules with *n*_1_ = *n*_2_ = *n*_3_ = 3. For a vector $v\in \{0,1{\}}^{n_{i}}$, we call every vector *x* ∈ {0, 1}^*d*^ for which for all *j* ∈ {1, …, *n*_*i*_} the $\left(\sum_{r=1}^{i-1}n_{r}+j\right)$-th component of *x* equals the *j*-th component of *v* an *i-extension* of *v*. For example in the SHM of Fig 1, the 9-dimensional vector (0, 0, 0, 1, 1, 0, 1, 0, 1) is a 1-extension of *v* = (0, 0, 0). We call *i*-extensions v*, w* of vectors $v,w\in \{0,1{\}}^{n_{i}}$ *equivalent* if v* and w* are component-wise equal except (possibly) for the components with indices in $\left\{\sum_{r=1}^{i-1}n_{r}+1,\ldots ,\sum_{r=1}^{i}n_{r}\right\}$. For instance, v* = (0, 0, 0, 1, 1, 0, 1, 0, 1) and w* = (1, 0, 0, 1, 1, 0, 1, 0, 1) are equivalent 1-extensions of *v* = (0, 0, 0) and *w* = (1, 0, 0) for the SHM of Fig 1. The idea behind this definition is that we want to extend inputs from single modules to inputs for the entire SHM.

#### Step 1: Conflict-graph construction

**Fig 3:**
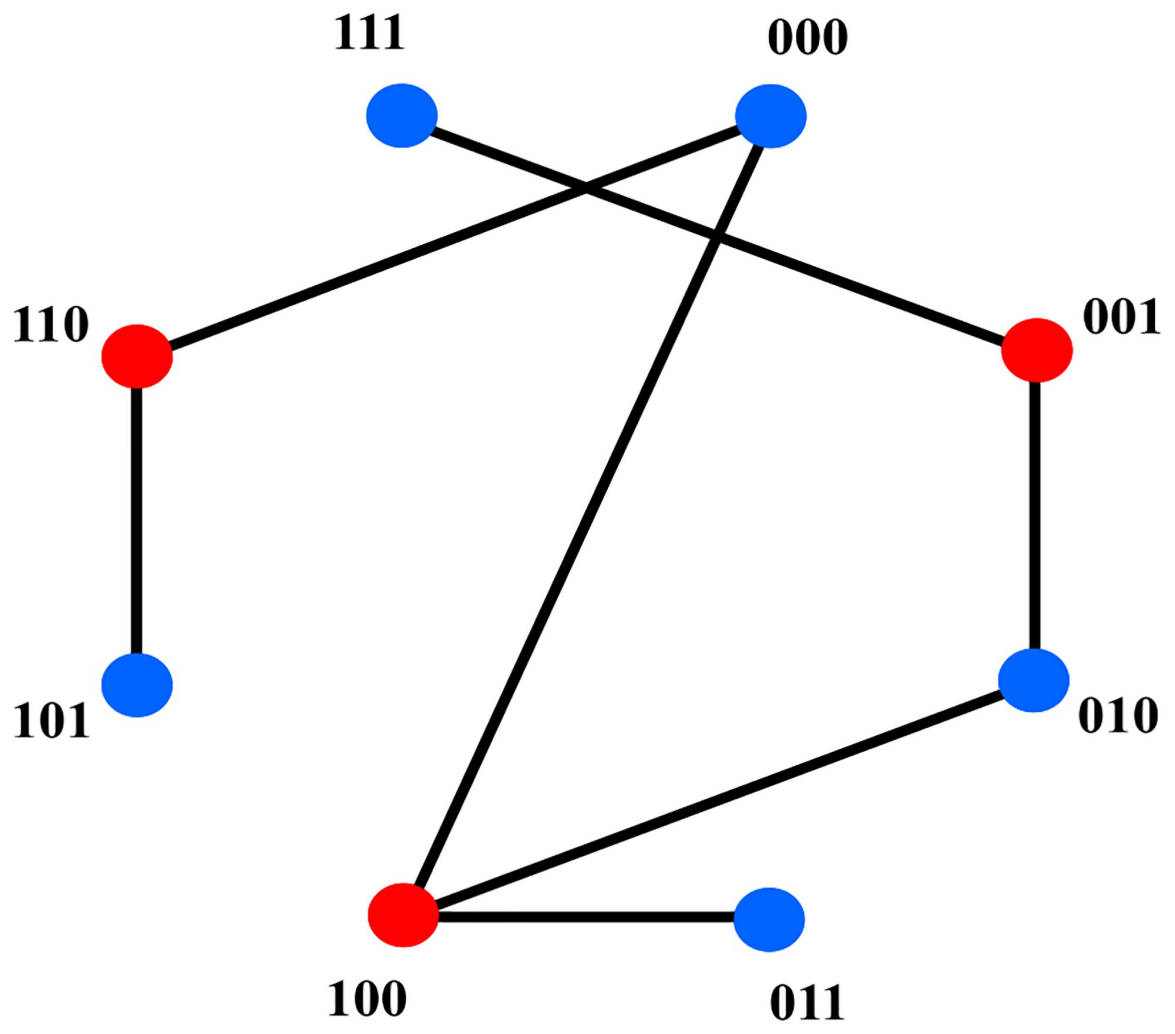
A 2-colorable graph representing the i-o function of a black-box module. Vertices of the graph denote all inputs of the module. Different colors of the vertices represent different outputs of the module. The graph has 8 = 2^3^ vertices because the module takes three binary variables as inputs.

In a binary classification setting, the i-o function of the *i*-th interior black-box module can be studied via a conflict graph *G*(*V*, *E*), see Fig 3. Here, the vertex set *V* corresponds to the set of all input vectors of the related black-box module. More precisely, the set *V* consists of the elements in $\{0,1{\}}^{n_{i}}$, where *n*_*i*_ is the number of input bits to the module. The set *E* is defined as follows: there is an edge between vertices *u* and *v* precisely if there exist equivalent *i*-extensions u* and v* of *u* and *v*, respectively, with different output labels.

Our learning strategy translates the information given by the training data into a conflict graph for every black-box module. For a graph *G*(*V*, *E*), the 2-coloring problem on *G* can be phrased as the task to find a mapping *f*:*V* → {0, 1} such that adjacent vertices always have distinct *f*-values. The partition of the vertex set into the two-color classes is unique if and only if the graph *G* is connected, i.e., there is a path between any two vertices in the graph [29]. By the definition of the edge set, the introduced conflict graphs must all be bipartite, i.e., 2-colorable. Indeed, consider an edge connecting two vertices *u* and *v* in the conflict graph for the *i*-th module *M*_*i*_. This means, by definition, that there are equivalent *i*-extensions of *u* and *v* which have different overall outputs. Since the *i*-extensions are equivalent, this change in the output can only be caused by different (intermediate) outputs of *M*_*i*_ on *u* and *v*. Hence, assigning to every vertex in the conflict graph for *M*_*i*_ the output of *M*_*i*_ on the corresponding *n*_*i*_-dimensional 0–1-vector constitutes a valid choice for *f*, proving the 2-colorability.

Suppose we are given a tree-SHM that maps binary input variables *x* ∈ {0, 1}^*d*^ to outputs *y* ∈ {0, 1} and has *k* first-layer black-box modules. For a set of training data vectors *x* with associated labels *y*, the steps of the Conflict-graph construction are as follows:

For *i* ∈ {1, …, *k*}, to construct the edges of the graph *G*_*i*_ of the *i*-th module, consider all pairs of vertices *v*, *w* ∈ *V*(*G*_*i*_) (i.e., all elements in $\{0,1{\}}^{n_{i}}$) and insert an edge between *v* and *w* precisely if there exist equivalent *i*-extensions v*, w* of *v* and *w* such that v* is labeled 0 and w* is labeled 1.

The 2-coloring problem of *G*_*i*_ has an unambiguous solution. In other words, the partition of the vertex set induced by the two colors is unique, if and only if *G*_*i*_ is a connected graph. In that case, we can determine the internal i-o function *f* of the *i*-th first layer module *M*_*i*_ up to a permutation of 0 and 1 by starting in an arbitrary vertex and assigning to it the value, say, 0. Then, by using a breadth-first-search, we can compute the partition of *V*(*G*_*i*_) into two sets $V_{i}^{0}$ and $V_{i}^{1}$, where for *j* ∈ {0, 1}, the set $V_{i}^{j}$ contains all inputs *x* to *M*_*i*_ with *f*(*x*) = *j*.

However, even if every graph *G*_*i*_ is connected, this only gives us information about the functions computed by the first-layer modules, i.e., for the intermediate outputs. To obtain the overall i-o function, we still need to combine this knowledge to compute the final outputs for all possible inputs. In the second step of our strategy, we, therefore, focus on determining actual output labels.

#### Step 2: Label determination

Having prepared the graphs *G*_*i*_ for all the modules, the Label determination aims to determine the unknown output labels of specific input data points by using the knowledge about the i-o functions of interior black-box modules acquired in Step 1. The determined labels for new input data points serve as new training data for Step 1 and may reduce the number of connected components in the updated *G*_*i*_, thus providing more information about the i-o function of the black-box modules. The Label determination applies the following well-known result due to Kőnig [30] to the bipartite graphs *G*_*i*_(*V*, *E*).

**Theorem.** *A graph G(V, E) is 2-colorable if and only if it has no cycles of odd length.*

Building on this insight, the following procedure tries to determine the labels of input data that are not yet labeled. Recall that for a tree-structured hybrid network with binary input vectors and *k* first-layer black-box modules, we can use a *k*-dimensional orthotope with cell labels to embody the i-o relation.

**I.** Let *x* be the lexicographically smallest *d*-dimensional binary vector for which the label has not yet been determined.
**II.** For *i* ∈ {1, …, *k*}, check if assigning label ‘0’/‘1’ to *x* creates a cycle of odd length in any of the *G*_*i*_ (by the definition of their edge relation). If it does, assign to *x* the opposite label, so as to maintain the 2-colorability of the graph by Kőnig’s Theorem.
**III.** If II was successful (i.e., a label was assigned) and there are still unlabeled input vectors, go to Step 1. If II was not successful and *x* was not the vector 1^d^, i.e., not the lexicographically largest vector, update *x* to the lexicographically next unlabeled input vector and repeat II. Otherwise, terminate.

**Fig 4:**
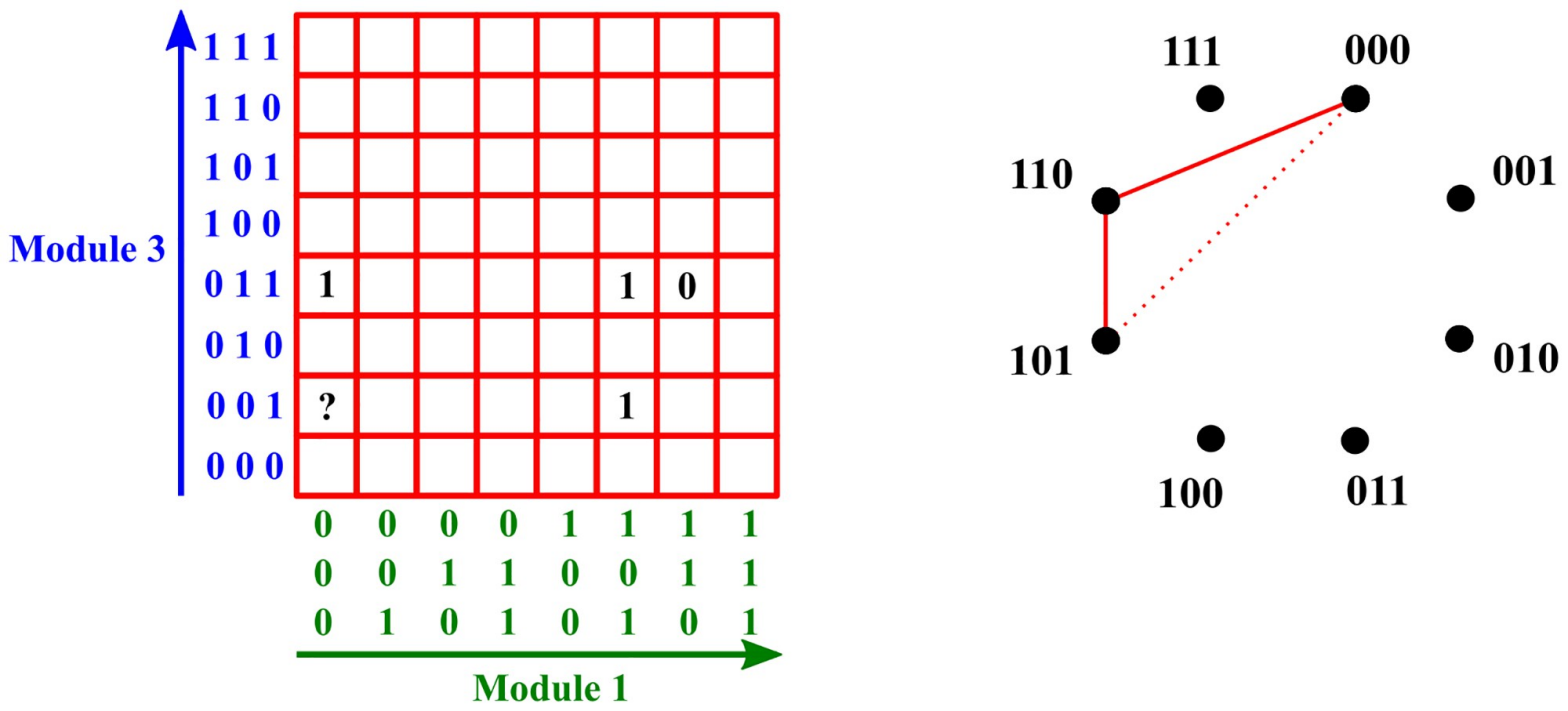
Schematic representation of the Label determination procedure. The left figure shows an intersection of a hyperplane of constant inputs, say 0, 0, 0, for Module 2 with the orthotope of the network of Fig 1 (i.e., a “red slice”). The right figure represents the related conflict graph for Module 1. In accordance with Kőnig’s theorem, adding the dashed line to the edge set breaks the bipartiteness of the graph. Since assigning label 0 to the input vector (0, 0, 0, 0, 0, 0, 0, 0, 1) would imply the existence of an edge between (0, 0, 0) and (1, 0, 1) in the conflict graph, the label for the ‘?’ cell must be 1.

Fig 4 gives a schematic representation of the Label determination procedure. By labeling previously unlabeled vectors, we expand the training data set for the Conflict-graph construction. Therefore, we can now repeat Step 1 and update the graphs *G*_*i*_ by inserting additional edges according to the new information. This way, alternating between Step 1 and Step 2, the procedure stops when we have filled the entire orthotope that states the labels for the possible input vectors or reached a situation where we cannot deduce further labels for the empty cells. The former case means that our learning strategy can extrapolate to the entire valid input space. In the latter case, however, additional training data points would be required to determine the labels of all unlabeled data points.

Algorithm 1 shows in pseudocode how the Conflict graph construction and the Label determination are combined to form our final algorithm.

**Algorithm 1** The full training strategy in pseudocode.

**Input:** A list of pairs (*x*, *ℓ*) of distinct data points *x* ∈ {0, 1}^*d*^ and binary labels ℓ ∈ {0, 1}; integers *n*_*i*_ for all *i* ∈ {1, …, *k*} (for some *k*) with $\sum_{i=1}^{k}n_{i}=d$

**Output:** A partial mapping *f*:{0, 1}^*d*^ → {0, 1} representing the label predictions that can be derived from the input data.

1: For every input (*x*, *ℓ*), set *f*(*x*)←*ℓ*.

2: **for** *i* ∈ {1, ⋯, *k*} **do**

3:  Initialize a graph *G*_*i*_(*V*_*i*_, *E*_*i*_) with $V_{i}=\{0,1{\}}^{n_{i}}$ and *E*_*i*_ = ∅.

4: **end for**

5: **While** *f* is not total **do**    //[i.e., there are *x* where *f*(*x*) is undefined]

6:  **for** *i* ∈ {1, ⋯, *k*} **do**

7:   **for** *v*, *w* ∈ *V*(*G*_*i*_) **do**

8:    **if** there are equivalent *i*-extensions v*, w* of *v*, *w* such that *f*(v*) = 0 and *f*(w*) = 1 **then**

9:     *E*_*i*_ ← *E*_*i*_∪{{*v*, *w*}}.

10:    **end if**

11:   **end for**

12:  **end for**

13:  *u* ← **false**    //*u* stores whether the following procedure updates *f*

14:  **for** *x* ∈ {0, 1}^*d*^ **do**

15:   **if** *f*(*x*) is undefined **then**

16:    **for** *i* ∈ {1, ⋯, *k*} **do**

17:     **if** setting *f*(*x*) ← 0 would create a cycle of odd length in *G*_*i*_ (by updating the edge set as described in Lines 7–9) **then**

18:      *f*(*x*) ← 1

19:      *u* ← **true**

20:     **else if** setting *f*(*x*) ← 1 would create a cycle of odd length in *G*_*i*_ **then**

21:      *f*(*x*) ← 0

22:      *u* ← **true**

23:     **end if**

24:    **end for**

25:   **end if**

26:  **end for**

27:  **if** *u* = **false then**

28:   **return** *f*

29:  **end if**

30: **end while**

31: **return** *f*

### Data sources

#### Synthetic data

To benchmark our learning strategy, we generated 30 tree structures for 2-layered SHMs with binary inputs *x* ∈ {0, 1}^*d*^, *d* ∈ {8, 9, 10, 11, 12}, three black-box modules in the first layer, and binary output labels *y* ∈ {0, 1}. The details and the schematic representation of the tree structures used for generating the synthetic data are depicted in S1 Appendix. Each structure was randomly constructed in a way that each first-layer black-box module operates on 2–6 separate input entries and forwards the partial results to the black-box output module.

We now describe how we obtained the labels for the synthetic data sets. For the associated SHM *H* of each data set, we generated random i-o functions for the three first-layer black-box modules and the output module. Then for each d-dimensional input to the network, we computed the partial outputs of the first layer followed by the overall output of the output layer, which we used as the label for the corresponding input vector.

#### COVID-19 data

This analysis was approved by the local ethical review board (EK 091/20; Ethics Committee, Faculty of Medicine, RWTH Aachen, Aachen, Germany). The Ethics Committee waived the need to obtain Informed consent for the collection, analysis of the retrospectively obtained, de-identified data as well as the publication of the results of the analysis. All methods were carried out in accordance with relevant guidelines and regulations.

Concerning the COVID-19 data, the studied population consists of patients with confirmed COVID-19 who had been admitted to the Intensive Care Unit (ICU) at University Hospital RWTH Aachen. The analyzed cohorts consisted of severely ill patients requiring invasive mechanical ventilation at least once throughout their ICU stay.

The clinical information of 63 adult patients (age ≥18 years) was collected between March and the end of June 2020. The median age was 62 years (interquartile range 58–70 years), and 66.7% of the patients (*n* = 42) were male. 27 patients did not survive during their ICU treatment, resulting in a mortality rate of 42.9%. The median length of stay in the ICU was 27.0 days (interquartile range 16.3—50.8 days). Table 1 presents the biometric and physiological parameters of the studied cohort of COVID-19 patients on the ICU admission, including the physiological parameters required for the sequential organ failure assessment score (SOFA score [31]).

**Table 1:** Biometric and physiological parameters of the studied COVID-19 patients on the ICU admission. Values are represented as n (%) or median (interquartile-range).

	Total	Survivor	Non-survivor
No.	63 (100)	36 (57)	27 (43)
Age (y)	62 (12)	60 (11.8)	66 (9.5)
Body mass index (BMI)	29.1 (5.9)	29.2 (4)	28.6 (11.5)
Male gender	42 (67)	26 (72)	16 (59)
Length of ICU stay (days)	27.0 (34.5)	34.8 (31.5)	17.6 (18.2)
Length of MV (days)	23.6 (30.6)	29.3 (31)	17.6 (17.9)
*PaO*_2_/*FiO*_2_ (mmHg)	92.4 (50.2)	99.9 (54.9)	88 (40.3)
Arterial pressure (mmHg)	56 (10.7)	56.6 (11.5)	54.5 (10)
Bilirubin	0.7 (0.5)	0.5 (0.4)	0.9 (1.1)
Platelets (×10^3^/*μl*)	220 (153)	215 (129.2)	241.5 (138)
Creatinine	1.1 (1.4)	0.9 (0.8)	1.3 (1.7)
Urine output (*ml*/*d*)	780 (1057.5)	800 (1245)	630 (870)

Almost all of the biometric and physiological patient information was collected as continuous values in diverse ranges and scales. Since our learning strategy requires binary data, we converted the initial continuous data into binary representations. As outlined in S2 Appendix, the first step of the data binarization was to use a decision-tree classifier to classify patients according to their vital status. We used biometric information and physiological parameters from the first seven days of the patients’ ICU stay as the attributes of the classification. S1 Table depicts the median and the interquartile range of the physiological parameters used in the decision-tree classifier. In the second step of the data binarization, we binarized the most important patient features obtained from the decision-tree classifier. The binarization threshold for each feature is its related critical value in the decision tree classifier. Table 2 shows these features and the critical values used for the binarization. Finally, we labeled the 5-dimensional binarized clinical patient data based on a 0.75 threshold on the mortality ratio. The obtained binary patient information and the associated mortality labels constitute the labeled data set for testing our learning strategy.

**Table 2:** Critical values for binarization of the most important COVID-19 patient features.

	‘0’	‘1’
Age (years)	<60	≥60
BMI-1	BMI < 26	BMI ≥ 26
BMI-2	(BMI < 24.2)∨(26 ≤ BMI < 32)	(24.2 ≤ BMI < 26)∨(BMI ≥ 32)
Acc.*(Min^‡^. ($\frac{PaO_{2}}{FiO_{2}}$)) (mmHg)	≥298	<298
Acc.(Min.(Urine output)) (ml)	<9584	≥9584

* $Acc(x)=\Sigma_{i=1}^{7}\frac{1}{7-i+1}x_{i}$ , where *x* is a sequence of 7 measurement values related to the first 7 days of ICU stay. The accumulation score sums up the values *x*_*i*_ of the days, with the days being weighted more heavily the closer they are to the respective point in time.

^‡^ A sequence of minimum values for each day of the ICU stay: *Min*(*x*) = [min(*x*_1_), …, min(*x*_7_)].

## Results and discussion

### Classification efficiency in the synthetic data

The main advantage of hybrid models compared with data-driven ones is the ability to extrapolate, i.e., to accurately predict labels for data points outside the convex hull of the given training data. In binary data, the convex hull of the training data only contains the training data itself. So in order for a binary classification algorithm to be meaningful, the extrapolation ability is crucial.

We summarize the extrapolability of our learning strategy on the synthetic data. We consider three different sizes *N*_tr_ of training-data sets containing 20%, 30%, and 40% of the whole data sets. For each training-data size, we sampled for each of the 30 SHM structures and for input dimensions *d* ∈ {8, 9, 10, 11, 12} five training-data sets of according size from the 2^d^ possible labeled data points. Then we executed our learning strategy and summarized the outcomes with five measurement results: classification accuracy, recall, precision, and F1-score [32]. Table 3 shows the results for the different training-data sizes *N*_tr_.

**Table 3:** Classification results of the hybrid model on the synthetic data.

*N*_*tr*_	Classification Result	Accuracy	Recall	Precision	F1-score
20%	Median (IQR*)	0.98 (0.03)	0.99 (0.05)	1.00 (0.02)	0.99 (0.03)
	Mean (SD^‡^)	0.93 (0.11)	0.93 (0.11)	0.97 (0.06)	0.95 (0.08)
30%	Median (IQR)	1.00 (0.01)	1.00 (0.00)	1.00 (0.00)	1.00 (0.00)
	Mean (SD)	0.98 (0.05)	0.97 (0.05)	0.99 (0.01)	0.98 (0.03)
40%	Median (IQR)	1.00 (0.00)	1.00 (0.00)	1.00 (0.00)	1.00 (0.00)
	Mean (SD)	0.99 (0.02)	0.99 (0.02)	0.99 (0.00)	0.99 (0.01)

* Interquartile range

^‡^ Standard deviation

For randomly chosen training-data sets containing at least 40% of the entire valid input space, the average of the classification accuracy is close to 1. In particular, for training data sizes *N*_tr_ of at least 40% of the entire valid input space, the median and the lower quartile of the classification accuracy are 1, and the mean of the classification accuracy is above 0.99. Furthermore, there exist (randomly sampled) training-data sets with classification accuracy equal to 1 even with *N*_tr_ equal to 20% of the entire valid input space. So the tree structure of the SHM suffices to guarantee the existence of small data sets with classification accuracy equal to 1. This property, which cannot be observed in pure ML methods, underlines that hybrid models have a high potential to reduce the training-data demand, see also [22, 23].

To compare the training data demand and classification efficiency of our method with other ML classifiers, we performed the same classification problem on the same synthetic data using different supervised learning methods, such as DNN, SVM, RF, and LR. We used grid-search cross-validation [33] as a hyperparameter tuning method for SVM, RF, and LR. In particular, we employed 5-fold stratified cross-validation on shuffled training data. The performances of the selected hyperparameters and trained models were then measured on a dedicated evaluation set that was not used during the model selection step. For DNNs, we used Keras Tuner [34, 35] hyperparameter optimization framework to optimize the hyperparameters of DNNs for each data dimension. The details of the optimized hyperparameters of the employed ML methods are shown in S3 Appendix.

**Fig 5:**
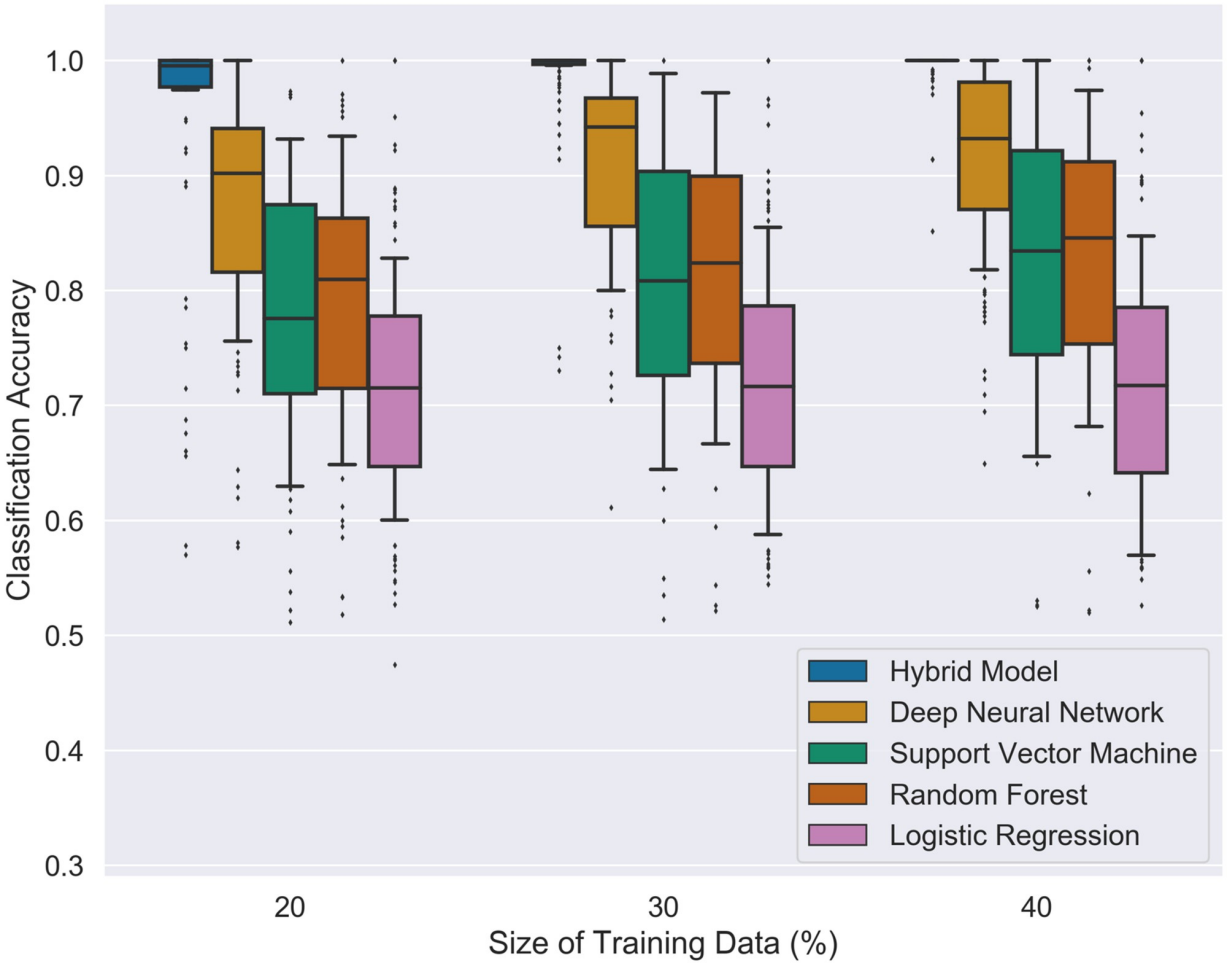
The distribution of classification accuracies for binary classification on the synthetic data. For each model and each size *N*_tr_ of the training data, we sampled 150 training data sets with input dimension *d* ∈ {8, 9, 10, 11, 12} and visualized the measured performance as box plots.

As summarized in Table 4, the classification efficiency of our hybrid model notably outperforms the other ML models, especially for smaller training-data sizes *N*_*tr*_. Fig 5 displays the increase in the classification accuracy when adding training data is much quicker in our classifier compared with the other models. In particular, the median of the classification accuracy of our strategy for training data sizes *N*_tr_ of at least 20% of the entire valid input space approaches 1, whereas, for the DNN classifier, it is still 0.93 for training data-sizes equal to 40% of the entire valid input space.

**Table 4:** The comparison of the median of the binary classification measurement results on the synthetic data.

*N*_*tr*_	Classifier	Accuracy	Recall	Precision	F1-score
20%	Hybrid Model	0.93	0.93	0.97	0.95
	Deep Neural Network	0.86	0.82	0.82	0.82
	Support Vector Machine	0.78	0.67	0.72	0.68
	Logistic Regression	0.72	0.55	0.61	0.56
	Random Forest	0.79	0.63	0.74	0.66
30%	Hybrid Model	0.98	0.97	0.99	0.98
	Deep Neural Network	0.90	0.87	0.85	0.85
	Support Vector Machine	0.80	0.71	0.75	0.72
	Logistic Regression	0.72	0.58	0.63	0.58
	Random Forest	0.81	0.67	0.77	0.70
40%	Hybrid Model	0.99	0.99	0.99	0.99
	Deep Neural Network	0.91	0.88	0.89	0.88
	Support Vector Machine	0.82	0.75	0.77	0.76
	Logistic Regression	0.72	0.57	0.63	0.58
	Random Forest	0.82	0.72	0.78	0.74

The superiority of our proposed methodology in the designed binary classification over the other supervised ML methods is firstly due to the reduced complexity in the SHM. An SHM employs various black-box modules with fewer input variables instead of a single black-box that deals with the entire input vector. The overall complexity of an SHM employing various black-box modules is usually much lower than an ML method, where a single black-box deals with the entire input vector. Secondly, our method can extrapolate, which is not the case for the other ML models. The extrapolability of our method boosts the classification performance of our methodology. As one of the consequences of the Curse of Dimensionality, the volume of the convex hull of *D*-dimensional data scales by 1/*D*! [36]. In an SHM, the union of the convex hulls of the sub-processes modules covers the volume that the hybrid model can make accurate predictions. As the volume of the union of the convex hulls of the sub-process is notably larger than the convex hull of the original data, 1/*d*_1_! + 1/*d*_2_! + … + 1/*d*_*k*_! ≪ 1/*D*!, where *d*_1_ + *d*_2_ + … + *d*_*k*_ = *D*, the binary classification performance of the hybrid classifier should outperform the other ML classifiers using a single black-box, which only guarantees faithful predictions inside the convex hull of the original data.

### Statistical analysis on the synthetic data classification results

We set up a statistical test for comparing the hybrid model with the other ML classifiers (DNN, SVM, LR, and RF) on the synthetic data. It has been shown that non-parametric tests are suitable for statistical comparisons of classifiers since they do not assume normal distributions or homogeneity of variance in accuracies or any other measure for the evaluation of classifiers [37]. In particular, the Friedman test with the corresponding post-hoc tests is recommended for comparing more than two classifiers over multiple data sets [37], which is the case in our problem.

The Friedman test is a non-parametric counterpart of the repeated-measures ANOVA [37, 38]. First, it separately ranks the algorithms for each data set according to their classification performances. Then it determines whether or not there is a statistically significant difference between the average ranks of the algorithms. The null-hypothesis *H*_0_ of the Friedman test states that all the algorithms are equivalent and so their ranks are equal. The Friedman statistic can be approximated by the Chi-squared distribution when the number of data sets *n* or the number of classifiers *k* is large enough (i.e. *n* > 15 or *k* > 4), which are the cases in our problem. For the significance level of *α* = 0.001 and the degree of freedom (the number of classifiers that we are comparing minus one) of DF = 4 the Chi-squared value equals 18.467. It means: if we calculate a Chi-squared value greater than the critical value of 18.467 in our test, then the null-hypothesis is rejected in favor of the alternative hypothesis *H*_1_ that the algorithms are not equivalent.

Table 5 shows the Friedman test results of comparing the five algorithms on 270 different data sets of our synthetic data using Statistical Tests for Algorithms Comparison (STAC) Python Library [39]. The resulted Friedman statistics or Chi-squared is 379.611 that rejects the null-hypothesis. Furthermore, the ranking of the algorithms is presented in Table 6 based on the average ranks of the algorithms over all data sets showing that our method is the best performing algorithm.

**Table 5:** The Friedman test with significance level of 0.001.

Friedman statistics	*P* value	Result
379.611	1.110 × 10^−16^	*H*_0_ is rejected

**Table 6:** The algorithms ranking.

Rank	Average rank on all data sets	Algorithm
1	1.372	Hybrid Model
2	2.281	Deep Neural Network
3	3.355	Random Forest
4	3.457	Support Vector Machine
5	4.533	Logistic Regression

We proceeded with the Holm method [40] as a post-hoc test to compare the ML classifiers with the hybrid model as a control model. The null-hypothesis in this case $H_{0}^{\prime }$ states that the control method is equivalent to the other algorithms (compared in pairs). The decision rule for rejecting the null-hypothesis is defines as whether the adjusted *P* value by the Holm method is lower than the significance level *α* = 0.001 or the test statistics *z* is greater than the critical value of 3.090 (for *α* = 0.001). The *z* value for comparing the *i*-th and *j*-th classifier is *z* $=|R_{i}-R_{j}|/\sqrt{(k(k+1)/6n}$, where *R*_*i*_ is the average rank of the *i*-th algorithm [37]. Table 7 shows the results of the post-hoc test using STAC Web Platform [39]. All the pairwise comparisons reject the null-hypothesis in favor of the alternative hypothesis $H_{1}^{\prime }$ that the control method, here the hybrid model, is not equivalent to the other algorithms.

**Table 7:** Post-hoc test using the hybrid model as the control method.

Comparison	*z* statistic	Result
Hybrid Model vs Support Vector Machine	15.322	$H_{0}^{\prime }$ is rejected
Hybrid Model vs Random Forest	14.574	$H_{0}^{\prime }$ is rejected
Hybrid Model vs Logistic Regression	23.229	$H_{0}^{\prime }$ is rejected
Hybrid Model vs Deep Neural Network	6.681	$H_{0}^{\prime }$ is rejected

We also investigated the influence of the dimensionality of the synthetic data set and the noise intensity in the classification efficiency of our model and compared the results with the other ML models. As the influences of the data dimensionality and the noise intensity in classification are highly dependent on the size of the training-data *N*_*tr*_, we performed the investigation for a fixed size of the training data, N = 200 data points (or patients), in a way that it is meaningful for clinical studies.

**Fig 6:**
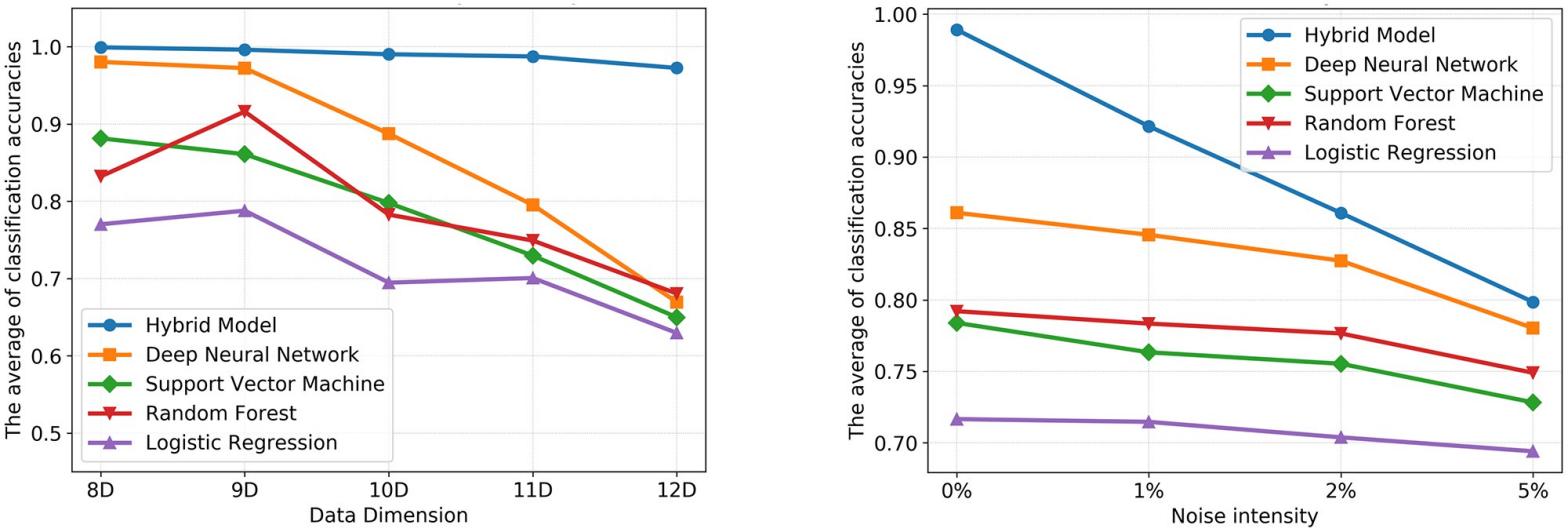
The influence of the dimensionality and the noise intensity of the synthetic data in the classification efficiency. The effect of data dimensionality (left) and noise intensity (right) in the average of the classification accuracy for 150 experiments executed for each model and for N = 200 data points.

The detailed results of the influence of the data dimensionality and noise intensity in the classification efficiency can be found in S4 Appendix, while visualization of the variation in model performance across different algorithms can be drawn from Fig 6. The classification efficiency of the hybrid model shows robustness against the increase of the data dimensionality, while, as attested by the COD, the performance of the other supervised ML algorithms notably suffers from the increase in the data dimension. Moreover, although adding noise to data can cause undesirable consequences to the prior knowledge that a hybrid model is built upon, the classification efficiency of the hybrid model outperforms other ML models even for noisy data.

**Fig 7:**
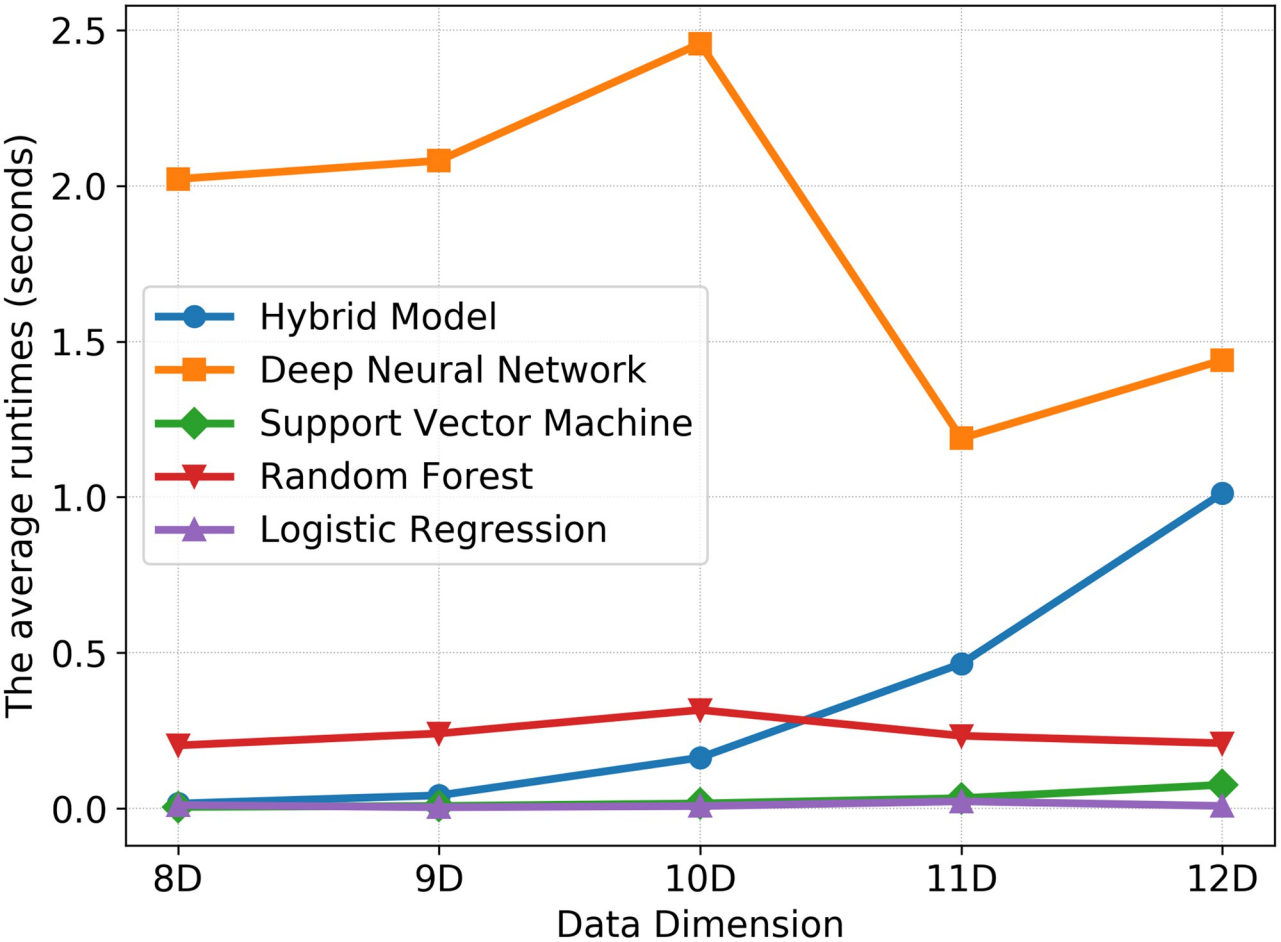
The running time efficiency. The comparison of the average running time of the examined methods for 150 experiments executed for each model and for N = 200 data points.

Lastly, we compared the time efficiency of our method with the other ML methods again with a fixed number of N = 200 training data points. Fig 7 shows the average running time for training the models and evaluating the predictions. The running time of the hybrid model is in the range of the other ML models even though the time needed for the hyperparameter optimization of the ML models is not considered.

### Mortality estimation for cohorts of COVID-19 patients

**Fig 8:**
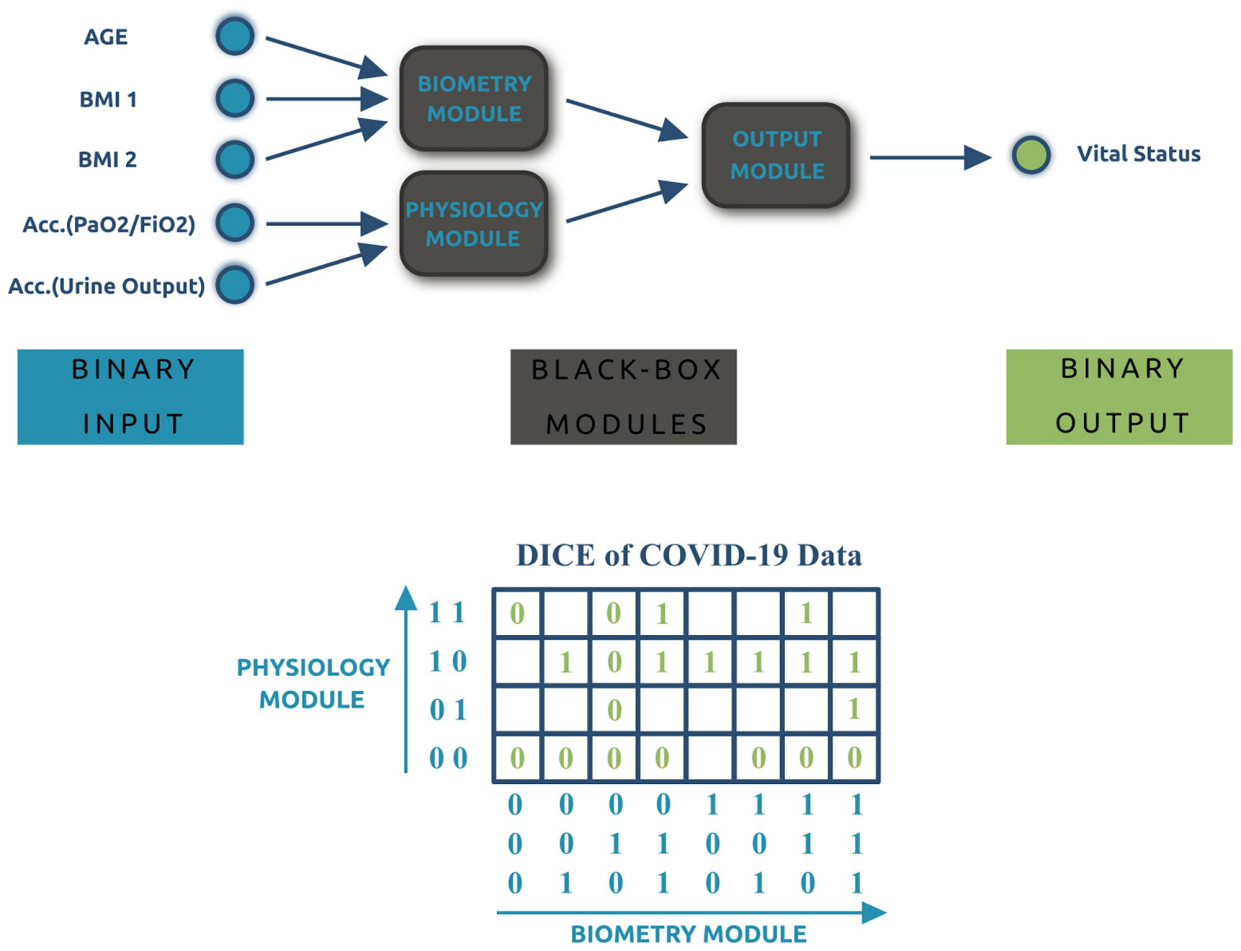
The SHM and the associated orthotope for the COVID-19 data. The upper figure shows the hybrid network mapping five binarized patient features to their vital status. The lower figure depicts the associated orthotope consisting of 20 cells with labels and 12 cells without a label.

To validate the potential applications of our strategy in life sciences, we studied the mortality in a cohort of 63 severely ill COVID-19 patients requiring ICU treatment. First, we fitted an SHM with an underlying tree structure to the COVID-19 data that maps the five binarized patient features (see Table 2) to the corresponding vital status. As discussed in S1 Appendix, the clinical and physiological information of the 63 patients was mapped to twenty different 5-dimensional binary representations out of the possible 2^5^. Based on the knowledge about the nature of the patient features in Table 2, we generated a hybrid network that consisted of two first-layer black-box modules, see the Biometric and Physiology modules in Fig 8. The Biometric module operates on the binarized age and BMI attributes of the patients. The Physiology module receives inputs related to the accumulation value of two physiological parameters, namely *PaO*_2_/*FiO*_2_ and the urine output. Fig 8 also illustrates the associated orthotope of the binarized COVID-19 data. The empty cells represent input data for which the label, i.e., the vital status, is still to be determined. The i-o functions obtained from the interior black-box modules of the COVID-19 hybrid network after training are shown in S1 Fig.

**Fig 9:**
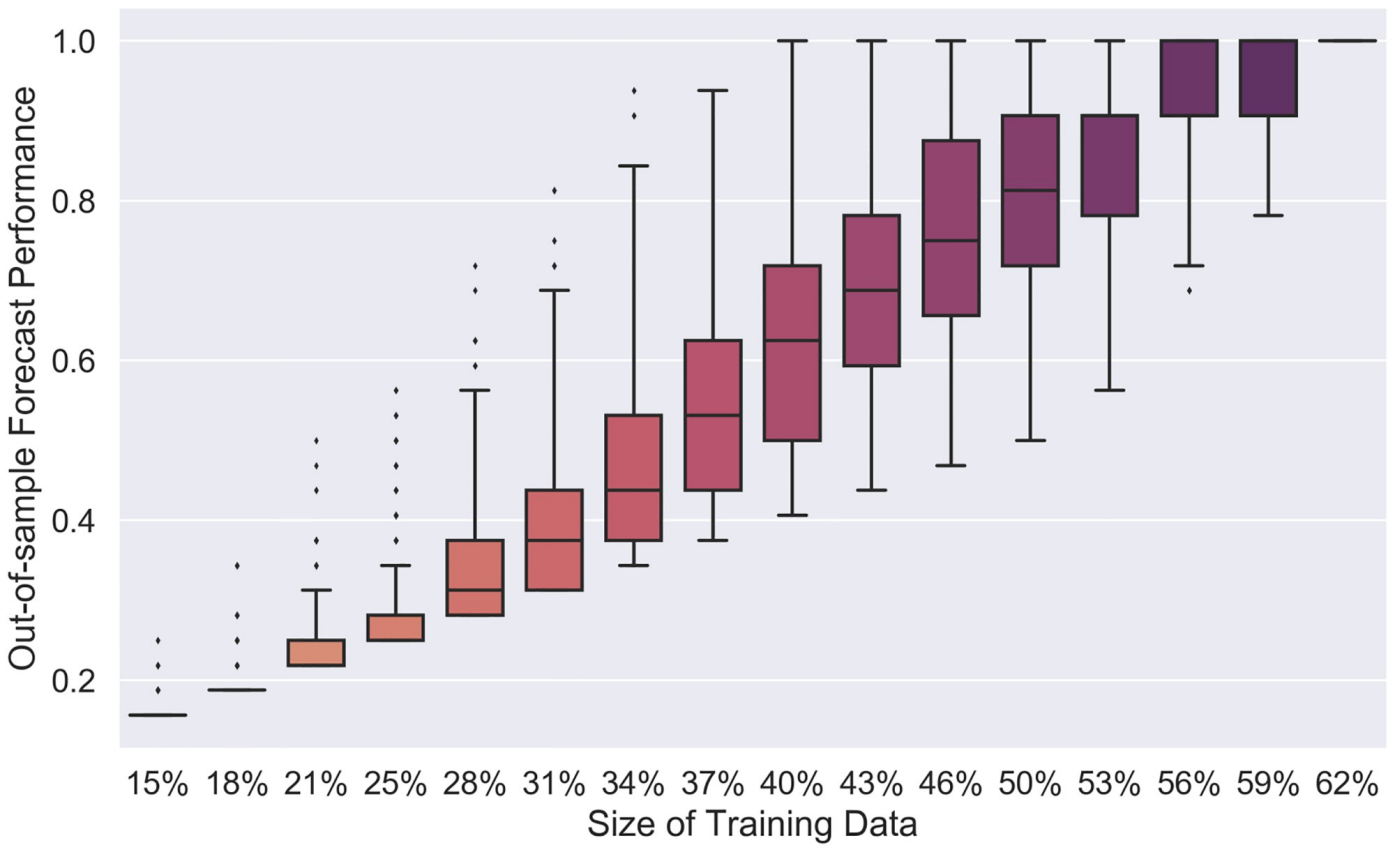
The out-of-sample forecast performance for the vital status of COVID-19 patients. The *x*-axis shows the percentage of the full input space that was used as the training data. For each training-data set size, we randomly sampled 1000 training data sets and measured the forecast performance. For randomly chosen training-data sets with a size of at least 56% of the entire valid input space, the median of the out-of-sample forecast performance equals 1.

Fig 9 illustrates the results of our learning strategy to predict the vital status of the patients. To cross-validate the results of our strategy, we partitioned the 20 available 5-dimensional binary representations of the patient data into a training and a validation set. The test-data set then consisted of the twelve unlabeled 5-dimensional binary representations of the patient data and the validation set. The *out-of-sample forecast performance* was calculated for randomly selected training data sets with 5 ≤ *N*_tr_ ≤ 20 for the tree-structured SHM. The out-of-sample forecast performance of an ML classifier is its test accuracy, which is the number of data points for which the label has been predicted correctly divided by the total size of the test-data set. Similarly, one can define the out-of-sample forecast performance of an SHM that performs a classification task as the number of unlabeled data points for which the SHM computes the right output divided by the total number of unlabeled valid inputs. The results on the COVID-19 data confirm the existence of training-data sets constituting ≈40% of the entire valid input space that has out-of-sample forecast performance equal to 1. Our method also yields out-of-sample forecast performance equal to 1 for all tested (randomly chosen) training-data sets of size at least 62% of the entire valid input space, which consists of the twenty different 5-dimensional binary representations of the considered 63 patients. The filled orthotope related to the COVID-19 hybrid network after training is shown in S2 Fig.

## Conclusion

In this paper, we proposed a learning strategy for binary classification tasks in which the classification can be computed by a tree-structured network with binary input vectors. We designed a structured hybrid model where the mechanistic knowledge about the system consists of the tree structure of the network that computes the output. The learning strategy is described for hybrid models with randomly distributed training data instead of densely distributed training data on low-dimensional manifolds as assumed in [22, 23], and thereby it can be considered a systematic extension of those works.

Compared with sole data-driven methods, our strategy promises a lower data demand since fewer parameters needs to be trained. As another direct result of incorporating prior knowledge into the modeling, it enables extrapolation. We evaluated our method by comparing its classification performance on synthetic data with various supervised ML algorithms. The numerical results testify to the lower data demand as well as the ability to extrapolate to the entire valid input binary space of our model.

We also applied our strategy to construct a tree-structured hybrid network that predicts the vital status of COVID-19 patients requiring intensive care-unit treatment and mechanical ventilation. The results show that our strategy can capture the mapping between binarized clinical patient information collected in the ICU stay and their vital status. As our application shows, the proposed learning strategy for training hybrid predictive models in clinical studies has the potential to extrapolate, i.e., make reliable predictions outside the convex hull of the given clinical data. This property can boost applications of ML in medical and clinical research where small-sized or biased clinical data sets occur.

There are two major limitations in this study that could be addressed in future research. First, the general method introduced in this paper is limited to binary input data. We are convinced that our method can be extended to continuous input data since the input to any network is always specified within a finite precision and can therefore be discretized and binarized. We plan to develop a proper data binarization step preparatory to the training step to handle this limitation. Second, the study focused on tree-structured networks. In a non-tree structured network, some input features are connected to more than one black-box module, which limits the Conflict-Graph construction part of our training strategy. One way to overcome this limitation is to omit those input features forming a non-tree structure and train the model with the remaining features for all possible combinations of the omitted features. However, this approach is not efficient in terms of training data demand. The generalization of the training strategy introduced in this paper to non-tree structured networks is a follow-up project that seems to require a heuristic approach.

## Supporting information

S1 Appendix Schematic representation of the tree structures. Tree structured networks mapping *x* ∈ {0, 1}^*d*^, where *d* ∈ {8, 9, 10, 11, 12}, to binary output labels *y* ∈ {0, 1}.
(PDF)

S2 Appendix COVID-19 data binarization. Conversion of the clinical and physiological COVID-19 patient features to binary variables.
(PDF)

S3 Appendix Hyperparameter optimization results. The optimized hyperparameters resulted from grid-search cross-validation and Keras tuner for the supervised ML methods.
(PDF)

S4 Appendix The influence of the dimensionality and noise intensity of the synthetic data on the classification efficiency. A comparison between the effect of data dimensionality and noise intensity on the classification efficiency of the hybrid model and the other supervised ML algorithm for the synthetic data.
(PDF)

S1 Fig The i-o functions obtained of the interior black-box modules of the COVID-19 hybrid network after training. (*Above:*) An overview of the i-o function of the Biometric module, the Physiology module, and the output modules of the COVID-19 hybrid network. The circular and the radial axes represent the binary inputs to the module and mortality rates for the considered 63 COVID-19 patients, respectively. The low mortality rates reflect noise in the patient data. (*Below:*) The active cells of the orthotope related to each black-box module are highlighted in blue.
(PDF)

S2 Fig Filled orthotope of the COVID-19 hybrid network. The filled orthotope of the COVID-19 network after performing the learning strategy. The black binary numbers represent the vital status in the original data, and the orange binary numbers display the predicted vital status.
(PDF)

S1 Table Physiological parameters used by the decision tree classifier of the COVID-19 patients’ vital status. The physiological parameters required for the SOFA score assessment. The parameters were evaluated for the first 7 days of ICU stay.
(PDF)
