# Supplementary material for: A training strategy for hybrid models to break the curse of dimensionality
Source: PLoS One. 2022 Sep 15;17(9):e0274569. doi: 10.1371/journal.pone.0274569 (PMC9477345; doi:10.1371/journal.pone.0274569)
Supplement: S1 Appendix — Tree structured networks mapping x ∈ {0, 1}d, where d ∈ {8, 9, 10, 11, 12}, to binary output labels y ∈ {0, 1}. (PDF) [file pone.0274569.s001.pdf]

S1 Appendix. Schematic representation of the tree structures

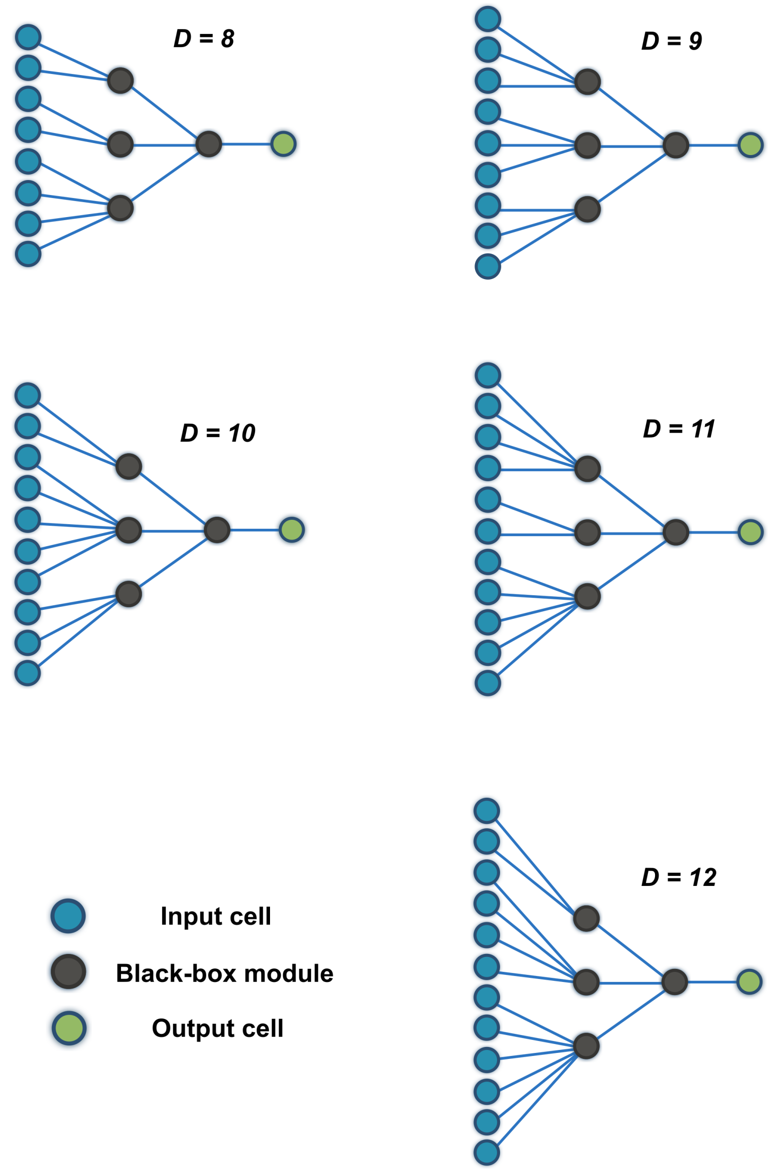

Figure 1: Tree structured networks mapping $x \in \{0,1\}^d$, where $d \in \{8, 9, 10, 11, 12\}$, to binary output labels $y \in \{0, 1\}$

In the above figure, some examples of the tree structure networks used for generating synthetic data are presented. All tree structures have 2 layers of black-box modules with binary inputs $x \in \{0,1\}^d$, $d \in \{8, 9, 10, 11, 12\}$, three black-box modules in the first layer, an output black-box modules in the second layer, and binary output labels $y \in \{0, 1\}$. Each structure was randomly constructed in a way that each first-layer black-box module operates on 2–6 separate input entries and forwards the partial results to the

black-box output module. For each input dimension, 6 different network structures had been created resulting in 30 tree structures in total. The table below shows the details of the connections of all the tree generated tree structures.

Table 1: **The connections between the input layer and the 3 black-box modules in the first layer of the structured hybrid models, named as n_1, n_2, and n_3.**

Input Data Dimension	n_1	n_2	n_3
8	2	2	4
	2	3	3
	2	4	2
	3	2	3
	3	3	2
	4	2	2
9	2	2	5
	2	3	4
	2	4	3
	2	5	2
	3	2	4
	3	3	3
10	2	2	6
	2	3	5
	2	4	4
	2	5	3
	3	3	4
	3	4	3
11	2	3	6
	4	3	4
	2	4	5
	3	3	5
	3	4	4
	4	4	3
12	2	4	6
	5	3	4
	2	5	5
	3	3	6
	4	4	4
	3	5	4
