# Supplementary material for: A training strategy for hybrid models to break the curse of dimensionality
Source: PLoS One. 2022 Sep 15;17(9):e0274569. doi: 10.1371/journal.pone.0274569 (PMC9477345; doi:10.1371/journal.pone.0274569)
Supplement: S2 Appendix — Conversion of the clinical and physiological COVID-19 patient features to binary variables. (PDF) [file pone.0274569.s002.pdf]

S2 Appendix. COVID-19 data binarization

Converting the clinical and physiological COVID-19 patient features to binary variables.

Step-1: Decision tree classifier

We studied biometric information and physiological parameters of the COVID-19 patients for the first 7-days of ICU stay. Figure 1 illustrates the results of a decision tree classifier for classifying patients according to their vital status. The decision tree classifier was implemented in Python using the open-source library Scikit-learn [1], and it was set to minimum-samples-split of 6 and minimum-impurity-decrease of 0.03 to avoid overfitting.

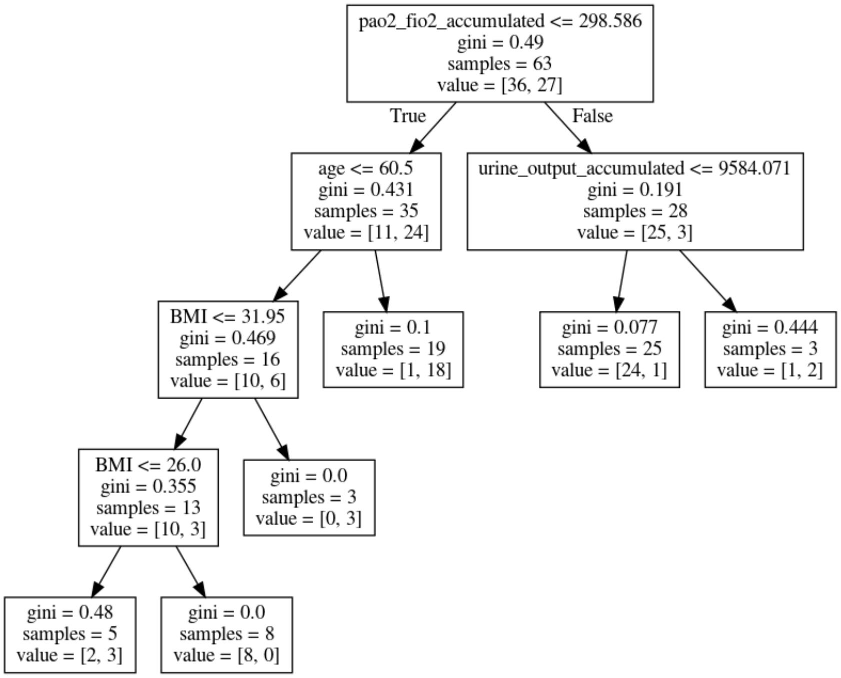

Figure 1: **Decision tree classification on the vital status of the COVID-19 patients.** 63 critically ill COVID-19 patients in which 27 were labeled as non-survivals were classified by a decision tree classifier using gini impurity criterion. The value quantities in the above figure represent [# survivals, # non-survivals] within the related sample size.

Step-2: Binarization

First, we binarized the five most important COVID-19 patient features using the critical values in the decision tree classifier. Then, a hybrid network with two different modules acting separately on the biometric information (Age, BMI) and the physiological parameters (PaO_2/FiO_2, Urine-output) was reconstructed, which maps 5-d binary input information to binary mortality status. Next, we labeled 5-d binarized COVID-19 patient clinical information based on a 75% cutoff on the mortality rates for the associated decimal repre-

sentation. As shown in Table 1, the information of 63 patients was embedded into 20 decimal configurations out of the possible 32.

Table 1: **Binary representation of COVID-19 patients features.**

Age	BMI-1	BMI-2	Acc.(PaO_2/FiO_2)	Acc.(Urine-output)	Decimal	Vital Status*
0	0	0	0	0	0	0
0	0	0	1	1	3	0
0	0	1	0	0	4	0
0	0	1	1	0	6	1
0	1	0	0	0	8	0
0	1	0	0	1	9	0
0	1	0	1	0	10	0
0	1	0	1	1	11	0
0	1	1	0	0	12	0
0	1	1	1	0	14	1
0	1	1	1	1	15	1
1	0	0	1	0	18	1
1	0	1	0	0	20	0
1	0	1	1	0	22	1
1	1	0	0	0	24	0
1	1	0	1	0	26	1
1	1	0	1	1	27	1
1	1	1	0	0	28	0
1	1	1	0	1	29	1
1	1	1	1	0	30	1

* Vital Status of ”0” and ”1” states survivals and non-survivals, respectively.

References

[1] Scikit-learn: Machine Learning in Python, Pedregosa et al., JMLR 12, pp. 2825-2830, 2011.
