# Supplementary material for: A training strategy for hybrid models to break the curse of dimensionality
Source: PLoS One. 2022 Sep 15;17(9):e0274569. doi: 10.1371/journal.pone.0274569 (PMC9477345; doi:10.1371/journal.pone.0274569)
Supplement: S3 Appendix — The optimized hyperparameters resulted from grid-search cross-validation and Keras tuner for the supervised ML methods. (PDF) [file pone.0274569.s003.pdf]

S3 Appendix. Hyperparameter optimization results

Here we show the optimized hyperparameters of the examined supervised learning ML classifiers, namely namely Deep Neural Network (DNN), Support vector Machine (SVM), Random Forest (RF), and Logistic Regression (LR), which is done by a grid search cross-validation object [1]. We employed 5-fold Stratified cross-validation on shuffled training data. The performances of the selected hyperparameters and trained model were then measured on a dedicated evaluation set that was not used during the model selection step.

1 Support Vector Machine

Table 1: The optimized hyperparameter of SVM for each input dimension of the synthetic data.

Input Data Dimension	C*	Kernel	Gamma**
8	10	rbf†	0.5
9	10	rbf	0.1
10	100	rbf	0.1
11	100	rbf	0.1
12	1000	rbf	0.1

* Regularization parameter

** Kernel coefficient

† Radial basis function Radial basis function

2 Random Forests

Table 2: The optimized hyperparameter of RF for each input dimension of the synthetic data.

Input Data Dimension	Bootstrap	Max. depth	Max. features	Min. samples leaf/split	# estimators
8	True	10	Auto	1/2	200
9	False	10	Auto	1/2	300
10	True	10	Auto	2/2	300
11	True	50	Auto	1/2	200
12	False	20	sqrt*	1/5	200

* Square-root of the number of features (here input data dimension)

3 Logistic Regression

Table 3: The optimized hyperparameter of LR for each input dimension of the synthetic data.

Input Data Dimension	C*	Penalty	Solver
8	0.001	none	Newton-cg
9	1	L1	liblinear
10	1	L2	Newton-cg
11	0.001	none	Newton-cg
12	0.1	L1	liblinear

* Inverse of regularization strength

4 Deep Neural Networks

For DNNs, we used Keras Tuner [2] hyperparameter optimization framework to optimize the hyperparameters of DNNs for each data dimensions. Here, we present the network summery of the networks related to each synthetic data input dimension.

For all different synthetic data input dimensions, we employed DNN classifiers with fully connected hidden layers. To compute the binary output of the network, we used a Softmax activation function. For training the DNN, we minimized the sparse categorical cross-entropy loss function of the predictions by the Adam's adaptive learning-rate optimization algorithm using the TensorFlow library [3].

Table 4: The network summery of the DNN employed for 8-dimensional synthetic data.

Layer type	Output shape	Activation function	# parameters
Dense	(None,48)	ReLu	432
Dense	(None,64)	Sigmoid	3136
Dense	(None,2)	Softmax	130

hidden layers = 1
Learning rate = 0.01
Total # trainable parameters = 3698

Table 5: The network summery of the DNN employed for 9-dimensional synthetic data.

Layer type	Output shape	Activation function	# parameters
Dense	(None,32)	ReLu	320
Dense	(None,48)	ReLu	1584
Dense	(None,2)	Softmax	98

hidden layers = 1
Learning rate = 0.001
Total # trainable parameters = 2002

Table 6: The network summery of the DNN employed for 10-dimensional synthetic data.

Layer type	Output shape	Activation function	# parameters
Dense	(None,48)	Tanh	528
Dense	(None,48)	Sigmoid	2352
Dense	(None,64)	Sigmoid	3136
Dense	(None,16)	Sigmoid	1040
Dense	(None,2)	Softmax	34

hidden layers = 3
Learning rate = 0.001
Total # trainable parameters = 7090

Table 7: The network summery of the DNN employed for 11-dimensional synthetic data.

Layer type	Output shape	Activation function	# parameters
Dense	(None,32)	Tanh	384
Dense	(None,48)	Tanh	1584
Dense	(None,48)	Tanh	2352
Dense	(None,32)	Tanh	1568
Dense	(None,2)	Softmax	66

hidden layers = 3
Learning rate = 0.01
Total # trainable parameters = 5954

Table 8: The network summery of the DNN employed for 12-dimensional synthetic data.

Layer type	Output shape	Activation function	# parameters
Dense	(None,48)	Tanh	624
Dense	(None,32)	Tanh	1568
Dense	(None,16)	Tanh	528
Dense	(None,2)	Softmax	34

hidden layers = 2
Learning rate = 0.01
Total # trainable parameters = 2754

References

[1] Scikit-learn: Machine Learning in Python, Pedregosa et al., JMLR 12, pp. 2825-2830, 2011.

[2] O'Malley T, Bursztein E, Long J, Chollet, F, Jin, H, Invernizzi, L. others: Keras Tuner. 2019, github.com/keras-team/keras-tuner.

[3] Abadi M, Agarwal A, Barham P, Brevdo E, Chen Z, et al. . Tensorflow: large-scale machine learning on heterogeneous distributed systems (2016). arXiv preprint arXiv:1603.04467. 2015;52.
