# Supplementary material for: A training strategy for hybrid models to break the curse of dimensionality
Source: PLoS One. 2022 Sep 15;17(9):e0274569. doi: 10.1371/journal.pone.0274569 (PMC9477345; doi:10.1371/journal.pone.0274569)
Supplement: S4 Appendix — A comparison between the effect of data dimensionality and noise intensity on the classification efficiency of the hybrid model and the other supervised ML algorithm for the synthetic data. (PDF) [file pone.0274569.s004.pdf]

S4 Appendix. The influence of the dimensionality and noise intensity of the synthetic data on the classification efficiency

Here we present a comparison between the effect of data dimensionality and noise intensity on the classification efficiency of the hybrid model and the other supervised ML algorithm for the synthetic data. As the influences of the data dimensionality and the noise intensity in classification are highly dependent on the size of the training-data N_{tr}, we perform the investigation for a fixed number of training data, N=200 data points (or patients), in a way that it is meaningful for clinical studies.

Table 1 presents classification measurement results, namely classification accuracy, recall, precision, and F1-score, for all examined methods and focuses on the effect of input data dimension on the classification efficiency of the methods.

Table 1: The average of the classification measurements of the examined methods on the synthetic data for different input data dimensions.

Input Data Dimension	Classifier	Accuracy	Recall	Precision	F1-score
8	Hybrid Model	0.99	0.99	0.99	0.99
	Deep Neural Network	0.98	0.94	0.97	0.95
	Support Vector Machine	0.88	0.84	0.82	0.82
	Logistic Regression	0.77	0.65	0.65	0.64
	Random Forest	0.83	0.67	0.78	0.70
9	Hybrid Model	0.99	0.99	0.99	0.99
	Deep Neural Network	0.97	0.97	0.96	0.95
	Support Vector Machine	0.86	0.78	0.82	0.80
	Logistic Regression	0.69	0.41	0.60	0.47
	Random Forest	0.91	0.83	0.89	0.85
10	Hybrid Model	0.99	0.98	0.98	0.98
	Deep Neural Network	0.88	0.84	0.85	0.85
	Support Vector Machine	0.79	0.71	0.73	0.72
	Logistic Regression	0.78	0.66	0.70	0.67
	Random Forest	0.78	0.56	0.79	0.64
11	Hybrid Model	0.98	0.97	0.98	0.98
	Deep Neural Network	0.79	0.75	0.76	0.75
	Support Vector Machine	0.72	0.65	0.67	0.66
	Logistic Regression	0.70	0.55	0.63	0.57
	Random Forest	0.74	0.61	0.70	0.65
12	Hybrid Model	0.97	0.98	0.97	0.97
	Deep Neural Network	0.66	0.63	0.64	0.63
	Support Vector Machine	0.64	0.59	0.62	0.60
	Logistic Regression	0.62	0.41	0.35	0.36
	Random Forest	0.68	0.62	0.64	0.61

Table 2 presents classification measurement results for three different noise intensities (1%, 2%, and 5%) for the synthetic data.

Table 2: The average of the classification measurements of the examined methods on the synthetic data for different noise intensities.

D*	Classifier	Noise intensity	Accuracy	Recall	Precision	F1-score
8	Hybrid Model	1%	0.96	0.97	0.97	0.96
		2%	0.89	0.93	0.91	0.91
		5%	0.83	0.88	0.84	0.83
	Deep Neural Network	1%	0.95	0.92	0.91	0.91
		2%	0.93	0.93	0.85	0.88
		5%	0.87	0.79	0.72	0.74
	Support Vector Machine	1%	0.85	0.76	0.78	0.76
		2%	0.84	0.76	0.77	0.76
		5%	0.81	0.65	0.71	0.67
	Logistic Regression	1%	0.77	0.60	0.59	0.58
		2%	0.73	0.55	0.61	0.58
		5%	0.74	0.53	0.55	0.54
	Random Forest	1%	0.83	0.66	0.77	0.68
		2%	0.82	0.64	0.80	0.68
		5%	0.78	0.56	0.72	0.61
9	Hybrid Model	1%	0.95	0.93	0.91	0.93
		2%	0.87	0.93	0.88	0.89
		5%	0.83	0.90	0.86	0.84
	Deep Neural Network	1%	0.94	0.92	0.93	0.92
		2%	0.93	0.91	0.89	0.90
		5%	0.88	0.84	0.83	0.84
	Support Vector Machine	1%	0.84	0.78	0.80	0.79
		2%	0.84	0.76	0.80	0.78
		5%	0.81	0.74	0.76	0.75
	Logistic Regression	1%	0.78	0.63	0.68	0.61
		2%	0.77	0.63	0.66	0.55
		5%	0.75	0.60	0.63	0.53
	Random Forest	1%	0.89	0.82	0.84	0.83
		2%	0.88	0.81	0.84	0.82
		5%	0.84	0.75	0.79	0.77

continued on the next page

D	Classifier	Noise intensity	Accuracy	Recall	Precision	F1-score
10	Hybrid Model	1%	0.89	0.89	0.90	0.89
		2%	0.86	0.87	0.89	0.86
		5%	0.76	0.74	0.81	0.75
	Deep Neural Network	1%	0.86	0.83	0.80	0.81
		2%	0.83	0.88	0.77	0.78
		5%	0.78	0.72	0.68	0.70
	Support Vector Machine	1%	0.74	0.65	0.65	0.63
		2%	0.73	0.63	0.65	0.64
		5%	0.71	0.62	0.62	0.62
	Logistic Regression	1%	0.69	0.56	0.64	0.59
		2%	0.69	0.54	0.61	0.56
		5%	0.68	0.52	0.62	0.55
	Random Forest	1%	0.77	0.54	0.77	0.62
		2%	0.76	0.57	0.75	0.63
		5%	0.74	0.48	0.74	0.56
11	Hybrid Model	1%	0.89	0.85	0.92	0.85
		2%	0.86	0.77	0.85	0.77
		5%	0.74	0.74	0.74	0.71
	Deep Neural Network	1%	0.77	0.74	0.71	0.71
		2%	0.76	0.71	0.71	0.71
		5%	0.69	0.69	0.61	0.62
	Support Vector Machine	1%	0.72	0.66	0.66	0.66
		2%	0.71	0.64	0.65	0.65
		5%	0.69	0.63	0.63	0.63
	Logistic Regression	1%	0.69	0.43	0.61	0.50
		2%	0.68	0.40	0.62	0.47
		5%	0.67	0.37	0.59	0.43
	Random Forest	1%	0.74	0.63	0.70	0.65
		2%	0.73	0.62	0.68	0.63
		5%	0.71	0.60	0.67	0.63
12	Hybrid Model	1%	0.89	0.87	0.91	0.88
		2%	0.78	0.70	0.72	0.69
		5%	0.71	0.67	0.67	0.63
	Deep Neural Network	1%	0.66	0.64	0.67	0.63
		2%	0.65	0.62	0.64	0.62
		5%	0.62	0.60	0.63	0.60
	Support Vector Machine	1%	0.64	0.59	0.61	0.60
		2%	0.64	0.60	0.59	0.59
		5%	0.60	0.57	0.58	0.57
	Logistic Regression	1%	0.62	0.43	0.40	0.44
		2%	0.62	0.47	0.35	0.43
		5%	0.61	0.43	0.30	0.35
	Random Forest	1%	0.67	0.60	0.64	0.59
		2%	0.66	0.60	0.63	0.58
		5%	0.65	0.59	0.61	0.57

* Input data dimension
