# Supplementary material for: A training strategy for hybrid models to break the curse of dimensionality
Source: PLoS One. 2022 Sep 15;17(9):e0274569. doi: 10.1371/journal.pone.0274569 (PMC9477345; doi:10.1371/journal.pone.0274569)
Supplement: S1 Fig — (Above:) An overview of the i-o function of the Biometric module, the Physiology module, and the output modules of the COVID-19 hybrid network. The circular and the radial axes represent the binary inputs to the module and mortality rates for the considered 63 COVID-19 patients, respectively. The low mortality rates reflect noise in the patient data. (Below:) The active cells of the orthotope related to each black-box module are highlighted in blue. (PDF) [file pone.0274569.s005.pdf]

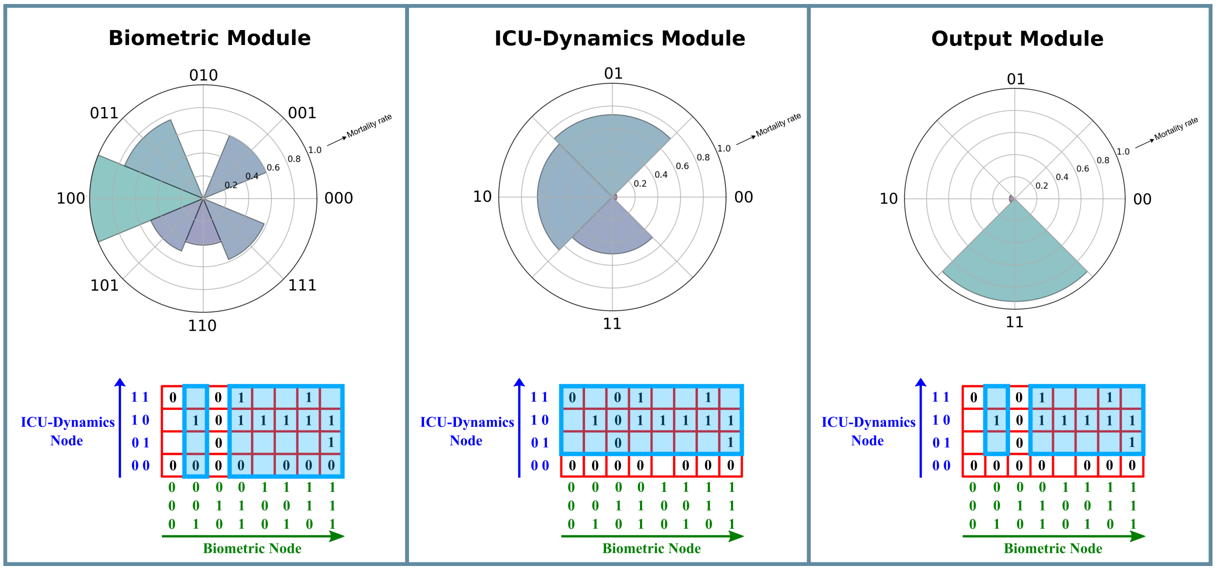

S1 Fig. The i-o functions obtained of the interior black-box modules of the COVID-19 hybrid network after training (*Above:*) An overview of the i-o function of the Biometric, the Physiology, and the output modules of the COVID-19 hybrid network. The circular and the radial axes represent the binary inputs to the module and mortality rates for the considered 63 COVID-19 patients, respectively. The low mortality rates reflect noises in the patient data. (*Below:*) The active cells of the orthotope related to each black-box module are highlighted in blue.
