# Supplementary material for: A training strategy for hybrid models to break the curse of dimensionality
Source: PLoS One. 2022 Sep 15;17(9):e0274569. doi: 10.1371/journal.pone.0274569 (PMC9477345; doi:10.1371/journal.pone.0274569)
Supplement: S2 Fig — The filled orthotope of the COVID-19 network after performing the learning strategy. The black binary numbers represent the vital status in the original data, and the orange binary numbers display the predicted vital status. (PDF) [file pone.0274569.s006.pdf]

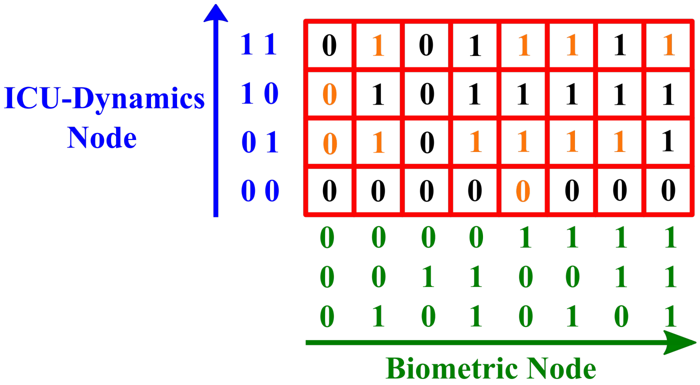


**S2 Fig. Filled orthotope of the COVID-19 hybrid network.** The filled orthotope of the COVID-19 network after performing the learning strategy. The black binary numbers represent mortality status of the original data, and the orange binary numbers demonstrate the extrapolated mortality status.
