# Supplementary material for: A training strategy for hybrid models to break the curse of dimensionality
Source: PLoS One. 2022 Sep 15;17(9):e0274569. doi: 10.1371/journal.pone.0274569 (PMC9477345; doi:10.1371/journal.pone.0274569)
Supplement: S1 Table — The physiological parameters required for the SOFA score assessment. The parameters were evaluated for the first 7 days of ICU stay. (PDF) [file pone.0274569.s007.pdf]

S1 Table. Physiological parameters used by the decision tree classifier of the COVID-19 patients' mortality status. The physiological parameters required for the SOFA score assessment. The parameters were evaluated for the first 7-days of ICU stay.

	**Total**	**Survival**	**Non-survival**
PaO_2/FiO_2 $(mmHg)$	109.92 (38.94)	124 (45.58)	96.01 (24.68)
Arterial pressure $(mmHg)$	54.5 (9.08)	55.21 (8.48)	52.85 (10.32)
Bilirubin (mg/dL)	0.66 (0.73)	0.55 (0.52)	1.02 (1.11)
Platelets $(\times 10^3/\mu l)$	255.57 (168.81)	259.14 (173.64)	241.42 (175.45)
Creatinine (mg/dL)	1.12 (1.28)	1 (0.72)	1.85 (1.54)
Urine output (ml/d)	2344 (2464)	2462 (1110)	1600 (2723)

Values are represented as median (IQR).
